# Inferring functional communities from partially observed biological networks exploiting geometric topology and side information

**DOI:** 10.1038/s41598-022-14631-x

**Published:** 2022-06-27

**Authors:** Jayson Sia, Wei Zhang, Edmond Jonckheere, David Cook, Paul Bogdan

**Affiliations:** 1 Ming Hsieh Department of Electrical and Computer Engineering, University of Southern California, Los Angeles, CA 90089 USA; 2 Department of Plant Pathology, Kansas State University, Manhattan, KS 66506 USA

**Keywords:** Bioinformatics, Plant hormones, Software

## Abstract

Cellular biological networks represent the molecular interactions that shape function of living cells. Uncovering the organization of a biological network requires efficient and accurate algorithms to determine the components, termed communities, underlying specific processes. Detecting functional communities is challenging because reconstructed biological networks are always incomplete due to technical bias and biological complexity, and the evaluation of putative communities is further complicated by a lack of known ground truth. To address these challenges, we developed a geometric-based detection framework based on Ollivier-Ricci curvature to exploit information about network topology to perform community detection from partially observed biological networks. We further improved this approach by integrating knowledge of gene function, termed side information, into the Ollivier-Ricci curvature algorithm to aid in community detection. This approach identified essential conserved and varied biological communities from partially observed *Arabidopsis* protein interaction datasets better than the previously used methods. We show that Ollivier-Ricci curvature with side information identified an expanded auxin community to include an important protein stability complex, the Cop9 signalosome, consistent with previous reported links to auxin response and root development. The results show that community detection based on Ollivier-Ricci curvature with side information can uncover novel components and novel communities in biological networks, providing novel insight into the organization and function of complex networks.

## Introduction

A key challenge in systems biology is understanding how individual molecular components interact to form functional communities giving rise to cell structure and function. Uncovering the organizing principles that determine these interactions is crucial for developing a systematic understanding of molecular functions of pathways and their crosstalk. One approach is to map the physical or functional interactions between cellular components, including DNA, RNA, proteins and small molecules, to construct a cellular biological network^1^. Such cellular biological networks represent biophysical snapshots of a global organization of cellular components. The maps of molecular interactions are often represented as networks, where nodes corresponding to cellular components are connected by edges representing either physical or functional interactions determined from the empirical interaction data^2–4^. A central hypothesis to biological network analysis is that highly connected nodes represent organized units, so called communities in network science, that conduct one or more biological functions. The level of functional organization can occur across scales, ranging from discrete motifs to larger order modules^5^. Conceptually, a motif represents the lowest functional organization, such that highly connected nodes perform a discrete function, while interacting motifs form modules that perform higher order cellular functions^6^.

Various community detection algorithms have been proposed to decompose complicated large-scale networks to infer their underlying organization and identify communities with shared functions. For instance, the Girvan–Newman (GN) algorithm^7^ iteratively removes the edge with the largest edge betweenness, a measure of the number of shortest paths passing through an edge, in a graph. Modularity-based community detection^8–10^ measures the quality of the division of the network into densely connected modules/communities. Although there are other community detection methods, i.e., spin model-based^11^ and random walk-based^12^ methods, the community detection for biological networks faces several challenges: (i) the high-dimensionality, complex multi-scale dynamics and interdependence among biological constituents renders current techniques as either inaccurate or provide incomplete understanding; (ii) community detection algorithms commonly applied to biological networks arose from the information theory and assume complete network information (i.e., all nodes and edges are known). This is rarely if ever true for biological datasets due to biological data acquisition conditions, such as specific developmental stages and growth conditions, and/or technological limitations. Consequently, only a partial snapshot of a complete molecular interaction network is available for data analysis, which may result in the missing nodes and/or node connections in a pathway^13–15^; (iii) related to network completeness, there is a trade-off between data quality and throughput, where efforts to capture more of a network’s nodes can suffer from higher false-positives, which will negatively impact community detection^16–18^; (iv) Current mathematical approaches do not integrate known biological node information during community detection. Known information is often used post-hoc to help identify community function, but the approach is often subjective and lacks a robust framework, failing to utilize the prior knowledge on biological functions of genes and pathways.

The applications of geometric analysis of networks have increased in the field of network science. Understanding the underlying topology of a network is important in revealing the salient properties of large-scale complex networks. One of the geometric network measures is the concept of network curvature. Ricci curvature and particularly its discretized forms^19^ (Ollivier-Ricci^20^ and Forman-Ricci^21^) have been applied in the study of networks. Earlier studies^22^ described the topological implications of curvature, particularly negative curvature, on the higher-order connectivity and the existence of central, influential neighborhoods in biological and social networks. Recent work using network Ollivier-Ricci curvature include the analysis of the internet topology^23^, quantifying the systemic risk and fragility of financial systems^24^, as well as cancer^25^, brain^26^, and drug-drug interaction^27^ networks and machine learning for biological applications^28^. The Forman-Ricci curvature has also been applied to the analysis of biological networks and its structures^29,30^. Additionally, the Ollivier-Ricci curvature has been applied as a tool for network community detection utilizing the implications of negative curvature^27^ and Ricci flow^31^. While conventional community detection algorithms rely on network structure alone, they ignore available valuable metadata, sometimes termed nodal attributes. Previous work extended the Stochastic Block Model to incorporate covariates or extra relevant information in network analysis^32,33^, and others incorporate nodal attributes as part of the community detection procedure^34–38^. Some of these not only allow community detection while considering additional nodal information, but are also able to generate networks with communities correlated with node attributes or deal with missing data^39,40^.

**Figure 1:**
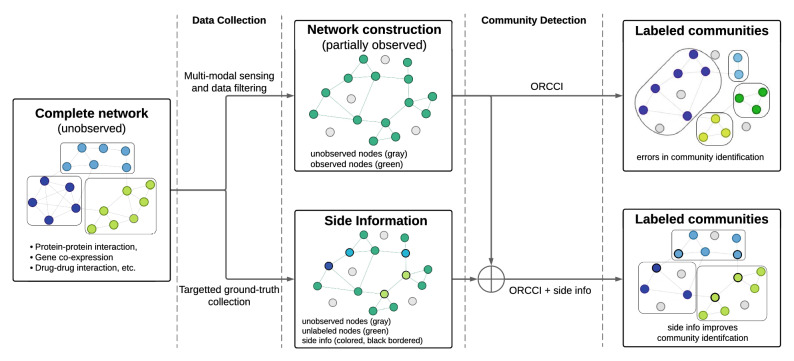
Community detection of partially observed networks utilizing geometric topology and side information. An idealized biological community is depicted on the left with three defined functional communities color coded and grouped. Experimental approaches are listed that attempt to capture this community, but complete node observation is often not achievable. The top row shows the typical process in biological network analysis: data collection, network construction, and community detection (based on the Ollivier-Ricci curvature community identification (ORCCI) algorithm). The bottom row depicts side community information typically available from expert knowledge or prior empirical analysis. Incorporation of this side information with community detection can improve community detection performance.

To further address the challenge of community detection in biological networks, we propose the Ollivier-Ricci curvature (ORC)-based community identification (ORCCI) coupled with side information to identify functional communities from graph representations of biological interaction data. Using this framework, we quantify the network geometric topology and incorporate prior knowledge about nodes into the community detection algorithm (Fig. 1). This ORC-based community detection analyzes the curvature of node connections (i.e., edges), iteratively removing edges and recomputing the curvatures until distinct communities are identified. The hypothesis is that edge curvature represents local community structure in a network, where positive ORC connects nodes in functional communities and negative ORC bridges between functional communities^27^. In addition to this novel algorithmic approach to community detection, our approach advances community detection by incorporating prior knowledge of node-level function annotation, so called side information (SI). Side information is inherently sparse for biological data, where most genes have an unknown function, but uses high-confidence functional annotation as ground-truth information for community function. We applied the ORC-based community detection coupled with side information approach on both synthetic and real-world protein-protein interaction (PPI) networks collected from various *Arabidopsis thaliana* experiments and evaluated the performance by comparing the functions of the detected communities with node-level ground-truth functions. The results demonstrate geometric topology and side information can be exploited to identify known functional communities, identify novel members of known and novel communities to understand complex biological networks.

## Results

### Side information improves ORC-based community detection performance in partially observed synthetic networks

**Figure 2:**
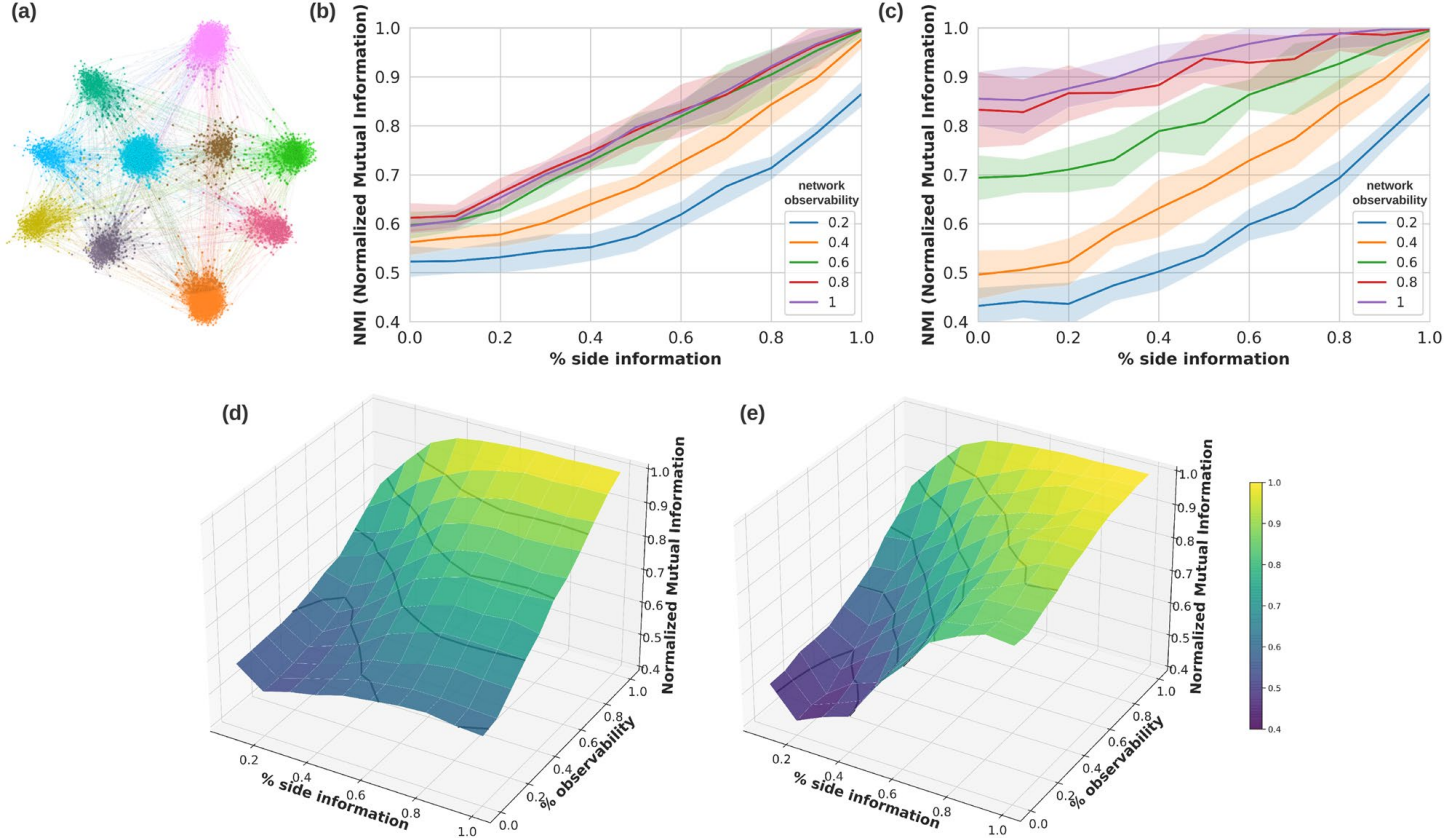
Partial network observation and side information analyses on synthetic networks. **(a)** A generated stochastic block model of network size 1000 with 10 pre-defined ground-truth communities visualized using ForceAtlas2 layout. Normalized mutual information (NMI) as a community detection performance criterion evaluated for the **(b)** ORC-final partitions and **(c)** ORC-maximum modularity partitions as a function of percentage side information for varying levels of network observations. Surface plots for all pairs of percentage side information and percentage observability values for **(d)** ORC-final partitions and **(e)** ORC-maximum modularity partitions.

To develop the geometric topology-based ORC community detection algorithm, we modified the ORC algorithm to include side information as a constraint during edge removal. Here, an edge that would normally have been removed, would be retained between two nodes if they shared the same side information. Our rationale was that adding known node information into biological network analysis would allow us to leverage prior experimental information and assist community detection. We analyzed the performance using synthetic networks constructed with the Stochastic Block Model (SBM)^41^. The synthetic networks contained 1000 nodes belonging to 10 pre-defined ground-truth communities (Fig. 2a). The community detection was performed over 100 iterations on the synthetic networks to calculate the average performance. To address how partial network observation impacts ORC-based community detection, we varied the percentage of observable nodes of the hypothetical networks from 20 to 100%. At the same time, we analyzed how side information (i.e., previously defined node function) impacts community clustering. We varied the amount of node-level side information for a graph from no side information ($0\%$) to full knowledge ($100\%$). Since the output of the ORC-based community detection is a hierarchical partition of the network, we considered two network partitions: ORC-final (denoted as *ORC*) is a fine-grained final partition of the network where all the negative curvature edges have been removed; and ORC-maximum modularity (denoted as *ORC MM*) is an intermediary partition of the network where the network modularity measure is maximized. As the *ORC MM* identifies communities prior to their terminal split, it results in merged and larger communities compared to *ORC*.

We used normalized mutual information (NMI) to evaluate ORC-based community detection performance on the synthetic networks^42^. For the *ORC* (final) partition, we found that increased network observability resulted in more accurate community detection in synthetic networks with a fixed amount of side information (Fig. 2b). Furthermore, increased network observability had a greater impact on community detection performance when more side information was known (0.6 versus 0.2 side information). This is likely because the limited network structure (i.e., fragmented connections) in a low observability setting (i.e., many missing nodes and therefore connections) impedes accurate community detection. Similarly, increasing the number of nodes with side information in synthetic networks with fixed network observability improved community detection performance (Fig. 2b). We found that side information had the greatest impact on community detection performance, where increased side information increased performance when network observability was in the middle test ranges (e.g., between 0.4 and 0.8). Interestingly, increasing network observability above 60% did not increase the community detection performance, regardless of the side information provided. This could be because even at non-complete observability, the synthetic network structure is strong enough that fewer tightly connected nodes represented near ground-truth communities. In addition, we did not observe complete community identification (NMI = 1) even having complete side information, especially at lower network observation ($< 0.4$). Lowering network observability can cause the loss of connections between smaller components in a known ground-truth community, which results in the breakup of the community into smaller “islands” or components even prior to community detection.

We next assessed the performance based on the *ORC MM* community partition. Similarly, increasing network observability and side information lead to more accurate community detection (Fig. 2c). With 0% side information, *ORC MM* had a lower NMI score of 0.44 than ORC partition of 0.52 at the same low network observability of 20%. However, *ORC MM* had a higher NMI score of 0.86 than *ORC* partition of 0.6 at full network observability of 100%. This is likely because maximum modularity works better for clear community structures with dense-connected community members^43^. The *ORC MM* results in wider standard deviation intervals, suggesting a higher volatility in the NMI score.

We integrated the varying levels of side information and network observability into surface plots for both the *ORC* and *ORC MM* to understand their impacts on community detection (Fig. 2d,e). Similarly, we observed that as the amount of side information decreased from 60% and below, the performance drops to a (min/max) NMI score between (0.6/0.85) to (0.5/0.6) and (0.6/0.95) to (0.45/0.85) for *ORC* and *ORC MM* partitions, respectively, and depending on the level of network observability. Overall, the side information improves the NMI score across all network observabilities for both ORC partitions. It is worth noting that regardless of the percent of network observability, having side information in the range of [0.2, 0.4] results in an increase in NMI performance. This shows that prior knowledge of community membership for certain subset of nodes can improve the discovery of network community.

**Figure 3:**
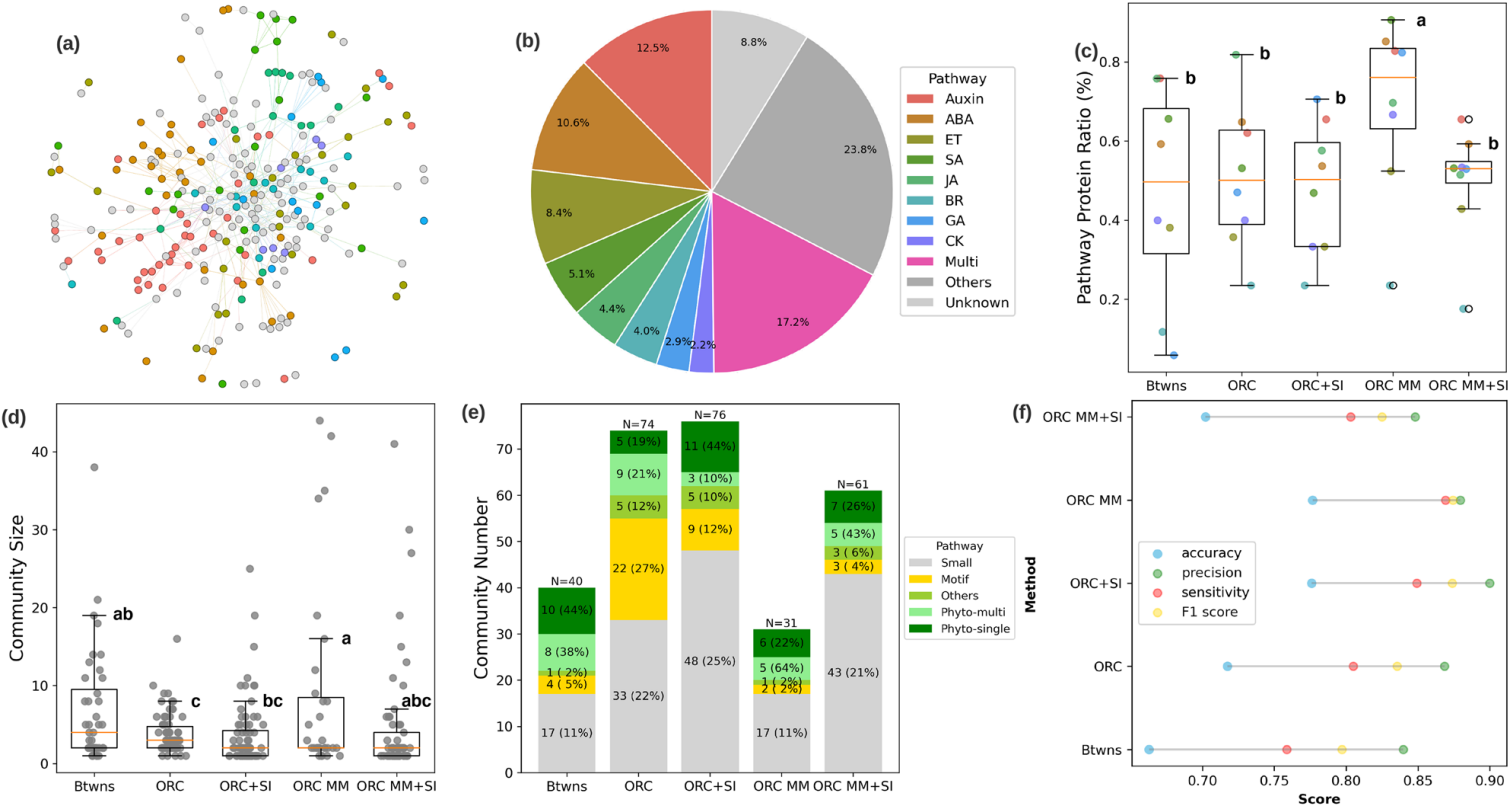
A comparison of network community detection methods for Arabidopsis phytohormone protein–protein interaction (PPI) $\hbox {PhI}_{{\mathrm{Main}}}$ network. **(a)** $\hbox {PhI}_{{\mathrm{Main}}}$ network with 273 nodes and 495 edges. Node colors represent known phytohormone pathway information (see legend in (**b**)). **(b)** Ratio of phytohormone pathway proteins compared to the entire network. Pathway labels: *Auxin* auxins, *ABA* abscisic acid, *ET* ethylene, *SA* salicylic acid, *JA*, jasmonic acid, *GA* gibberellic acids, *BR* brassinosteroids, *CK* cytokinins, *Multi* multi-labeled pathway protein, *Others* other non-phytohormone pathways and other associated gene function (e.g., transcription factor, transporter, stress, and abiotic-biotic stimulus); and, *Unknown* unlabeled proteins. Different community detection (CD) methods are compared with **(c)** percentage of identified pathway protein ratio, **(d)** community size distribution and (**e)** community number differentiated according to small, motif, single- or multi-function phytohormone, or other non-phytohormone pathway communities. Method labels: Btwns, Betweenness^44^, the baseline method; *ORC*, Ollivier-Ricci curvature-based method^27^ (at final partition); *ORC+SI*, ORC with side information; *ORC MM*, ORC (at maximum modularity partition); *ORC MM+SI*, ORC MM with side information. Boxplot labels are obtained from the Turkey post hoc test for multiple comparisons. Community functions are determined by the Kyoto Encyclopedia of Genes and Genomes (KEGG) analysis^45^, Gene Ontology (GO) enrichment analysis^46^, and annotated phytohormone pathway proteins. **(f)** Performance comparison of the different methods in terms of accuracy, precision, sensitivity and F1 score.

### Ollivier-Ricci curvature and side information improve community detection from a small Arabidopsis protein network

To evaluate the performance of ORC-based community detection coupled with side information on real-world biological networks, we applied the developed method to an Arabidopsis PPI dataset. We first tested whether the ORC-based community detection methods could identify functional communities from a subset of the Arabidopsis phytohormone signaling interactome, which the original authors termed $\hbox {PhI}_{{\mathrm{MAIN}}}$^47^. The $\hbox {PhI}_{{\mathrm{MAIN}}}$ sub-network was developed from the full Arabidopsis phytohormone signaling interactome dataset ($\hbox {PhI}_{{\mathrm{FULL}}}$) and cross-referenced against two literature-based^48,49^ networks to improve the false discovery rate. After curation, the $\hbox {PhI}_{{\mathrm{MAIN}}}$ network contains 495 binary interactions among 273 proteins (Fig. 3a). From the total proteins, 67.3% of them contained functional annotation belonging to at least one of the eight phytohormone pathways, including auxin, abscisic acids (ABA), ethylene (ET), salicylic acids (SA), jasmonic acids (JA), gibberellic acids (GA), brassinosteroids (BR), and cytokinins (CK) (Fig. 3b). These phytohormone labels were used as node-level side information to aid the ORC-based community detection (colored nodes in Fig. 3a and Supplemental Table 1). We evaluated how the two ORC-based community detection network partition levels, together with and without side information, (*ORC*, *ORC+SI*, *ORC MM*, *ORC MM+SI*), performed and benchmarked the results versus an edge betweenness-based^7^ community detection algorithm previously applied to the $\hbox {PhI}_{{\mathrm{MAIN}}}$ network^47^. From the four ORC-based community detection approaches, we identified 18–30 potential functional communities from $\hbox {PhI}_{{\mathrm{MAIN}}}$. To address if these communities represent the known phytohormone pathways, we labelled each community using Kyoto Encyclopedia of Genes and Genomes (KEGG) pathway analysis^45^ and Gene Ontology (GO) enrichment analysis^46^ (Supplemental Table 2). For communities with phytohormone labels, we measured the ratio of nodes with a matching phytohormone annotation to the total nodes in the community, termed pathway protein ratio (Fig. 3c). The ORC-based community detection results for *ORC*, *ORC+SI* and *ORC MM+SI* had an average pathway protein ratio approximately 50% and the *ORC MM* exhibited the highest average ratio at 70% (Fig. 3c). These results were either comparable or better than the previous edge betweenness method (*Btwns*) (Fig. 3c), which suggests that the ORC-based community detection coupled with side information can cluster phytohormone proteins into their corresponding biological communities.

The community size distributions for *ORC* and *ORC+SI* showed a majority of communities of sizes 10 and below compared to *ORC MM* and *ORC MM+SI* showed some communities of sizes greater than 25 (Fig. 3d). This tallies with the expectation that *ORC MM* results in larger sized communities. Conceptually, nodes are hierarchically organized to provide cellular functions into motifs (smallest functional units with less than five interacting proteins) and modules (larger functional groups of interacting motifs and proteins)^5^. We found that the control edge-betweenness method led to fewer small communities (size $\le 3$) and motifs ($3<$ size $\le 5$) compared to all four ORC-based approaches, but identified more modules (size $> 5$), representing communities made up of multiple labels. The ORC final partition methods (*ORC* and *ORC+SI*) identified a large proportion of small communities and motifs (Fig. 3e). For example, *ORC+SI* identified 11 single-function phytohormone modules compared to 5 such modules by *ORC*, and only 3 multi-function phytohormone modules compared to 9 such modules by *ORC* (Fig. 3e). Side information can help *ORC* final partition to merge communities with shared functions into a larger module and break a multi-function module into several small single-function motifs and modules. The *ORC MM* identified more large-size modules and fewer small communities and motifs (19 communities) compared to the *ORC* final partitions (55 for *ORC*, and 57 for *ORC+SI*) (Fig. 3e). By integrating protein phytohormone pathway side information, the *ORC MM+SI* can breakdown the giant modules with mixed functions (phyto-multi) into several modules with distinct functions (phyto-single) (Fig. 3d,e). These results show how side information improves the identification of single-function communities. The side information can help recognize the neighborhoods with shared functions, which are usually detected as separate communities by *ORC* as this algorithm is executed until there are no more valid partition splits according to the ORC community partition rule. Thus, *ORC+SI* identifies more modules with smaller size and fewer number of total motifs and modules, which facilitate the downstream study^45,46^ of the biological function(s) of a specific module (Fig. 3d,e). *ORC MM+SI* did not help improve the average phytohormone pathway protein ratio because the maximum modularity partition tends to give larger community partitions by merging two or more small phytohormone communities due to their relatively close connectivity (Fig. 3d), which resulted in a biased higher average phytohormone pathway protein ratio. *ORC MM+SI* can help breakdown the merged community into small communities with dedicated functions as can be seen from the lower mean community size (Fig. 3d) and increased number of communities (from $N=31$ to 61) (Fig. 3e) compared to *ORC MM*.

The results where additionally assessed using standard information retrieval criteria^50^, where we found that *ORC+SI* gave the best results, and the four ORC-based methods resulted in better classification performance than the *Btwns* method (Fig. 3f). We found that side information improved ORC performance, (i.e., *ORC+SI* has higher accuracy, precision, sensitivity and F1-score compared to *ORC*). However, *ORC MM+SI* had lower performance compared to *ORC MM*. The seemingly reduced performance when side-information was added to *ORC MM* was attributed to the way KEGG/GO assigns multiple functions to large communities inflating the performance score. With side information, this has led to more smaller communities with dedicated functions (Fig. 3f). Taken together, ORC-based community detection coupled with node side information can detect physical and functional motifs and modules in the Arabidopsis $\hbox {PhI}_{{\mathrm{MAIN}}}$ network.

It is important to note the influence that SI has on correct community detection in our experiments. As argued in Ref.^51^, metadata and ground truth are not the same. We sought to minimize the influence of incorrect SI in the current work by using carefully curated SI based on high-confidence functional annotation from prior literature^45,46,52,53^. To directly assess how incorrect side-information impacts community detection, we compared our community detection results to those obtained when varying levels of incorrect side-information were present. Here, we randomly assigned an increasing percentage of nodes in the a network with incorrect SI (see Supplementary Information for details). The results show that as SI in the network becomes increasingly incorrect, the quality of the community detection, as measured by accuracy for instance, become increasingly worse (Fig. S6).

**Figure 4:**
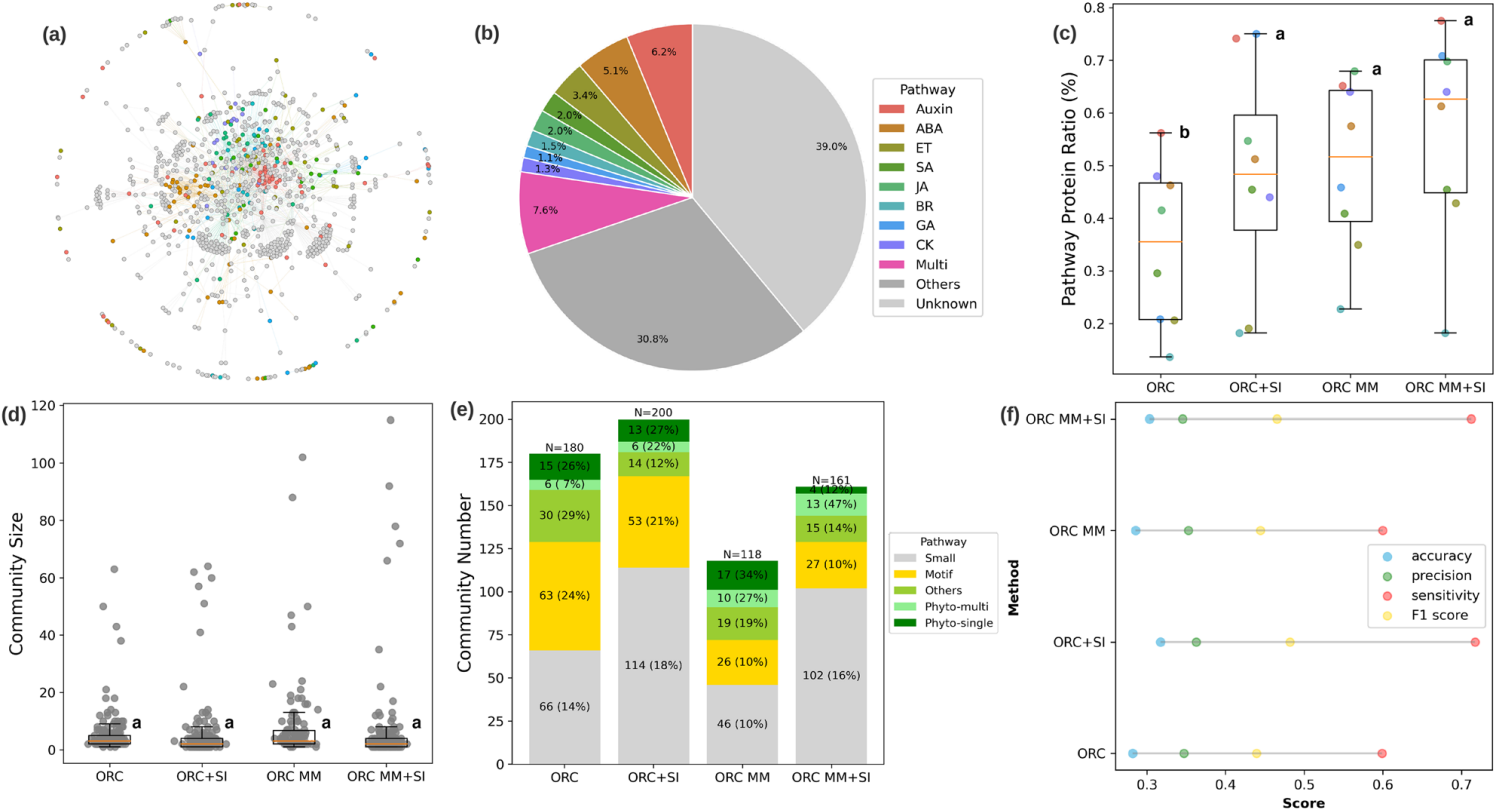
A comparison of network community detection methods for Arabidopsis phytohormone protein–protein interaction (PPI) $\hbox {PhI}_{{\mathrm{Full}}}$ network. **(a)** Network visualization with 925 nodes and 2021 edges. Node colors represent known phytohormone pathway information (see color legend in **(b)**). **(b)** Ratio of phytohormone pathway proteins compared to the entire network. The different community detection (CD) methods are compared with **(c)** percentage of identified pathway protein ratio, **(d)** community size distribution, and (**e)** community number. **(f)** Performance comparison of the different methods in terms of accuracy, precision, sensitivity and F1 score. See Fig. 3 for pathway and method labels.

### Side information improves community detection performance for the expanded Arabidopsis phytohormone network

To test the performance on biological networks with increased size and complexity, we next applied the ORC-based community detection approaches (*ORC*, *ORC+SI*, *ORC MM*, *ORC MM+SI*) to the full phytohormone interactome dataset, termed $\hbox {PhI}_{{\mathrm{FULL}}}$. Compared with the curated $\hbox {PhI}_{{\mathrm{MAIN}}}$ network, the expanded $\hbox {PhI}_{{\mathrm{FULL}}}$ network was suitable for exploration of local network topological structures and hypothesis generation on novel components in a biological pathway and crosstalk among pathways^47^. The $\hbox {PhI}_{{\mathrm{FULL}}}$ PPI network contains 2021 binary interactions among 925 proteins, which is roughly 4$\times$ more interactions and over 3$\times$ more proteins than the $\hbox {PhI}_{{\mathrm{MAIN}}}$ dataset (Fig. 4a). Only 30.2% of $\hbox {PhI}_{{\mathrm{FULL}}}$ proteins belong to the eight main phytohormone pathways, 30.8% belong to other biological pathways, and 39.0% are unknown proteins without functional annotation, which makes this dataset substantially more difficult to identify the phytohormone communities due to the potentially increased noise and more limited node-level side information (Fig. 4b). For the $\hbox {PhI}_{{\mathrm{FULL}}}$ network, we used side information for $22.6\%$ of the 925 proteins (colored nodes in Fig. 4a), compared to the 50.1% of 273 proteins that had side information for the $\hbox {PhI}_{{\mathrm{MAIN}}}$ network analysis. The betweenness-based community detection was not calculated for the $\hbox {PhI}_{{\mathrm{FULL}}}$ network, and we therefore evaluated the performance among the four ORC-based community detection methods. Only with the assistance of side information, the two ORC-based methods (*ORC+SI*, *ORC MM+SI*) show a higher average pathway protein ratio on the $\hbox {PhI}_{{\mathrm{FULL}}}$ network (Fig. 4c) compared to their performance on the $\hbox {PhI}_{{\mathrm{MAIN}}}$ network. *ORC+SI* identified less motifs (53) and modules (33) in the $\hbox {PhI}_{{\mathrm{FULL}}}$ network (Fig. 4e) compared to the *ORC* method (63 motifs and 52 modules, respectively) which is a similar observation in $\hbox {PhI}_{{\mathrm{MAIN}}}$ network. Although side information helps *ORC MM* breakdown the largest communities, we did not find more single-function modules identified by *ORC MM+SI* than *ORC MM*, which was different from the *ORC MM+SI* performance on $\hbox {PhI}_{{\mathrm{MAIN}}}$ network (Fig. 4d,e and Supplemental Table 4). The drop in the ability to identify single-function modules by *ORC MM+SI* may be due to the increased network size and number of interactions among proteins in $\hbox {PhI}_{{\mathrm{FULL}}}$ network. In agreement with this observation, we found more large modules of size greater than 60 that were detected by the four ORC-based approaches. The increase in network size resulted from the incorporation of unknown labelled genes into known functional communities (Fig. 4d, Supplemental Table 4). In particular, *ORC+SI* detects more biological meaningful modules than the other three ORC-based methods, including 19 modules with phytohormone functions (phyto-single and phyto-multi) and 14 modules with other functions, such as gene regulation, protein processing, and RNA processing (Fig. 4e). The global evaluation revealed the use of side information can slightly improve community detection performance for both *ORC* and *ORC MM* in $\hbox {PhI}_{{\mathrm{FULL}}}$ network (Fig. 4f). The increased complexity, attributed by the increase in network size and number of interactions as well as fewer side information, resulted in a drop in performance on the $\hbox {PhI}_{{\mathrm{FULL}}}$ network compared to the $\hbox {PhI}_{{\mathrm{MAIN}}}$ network. Taken together, ORC-based community detection can still identify the biological meaningful communities in a more complicated protein network, and the side information can improve the community detection performance despite the even smaller set of nodes with side information.

**Figure 5:**
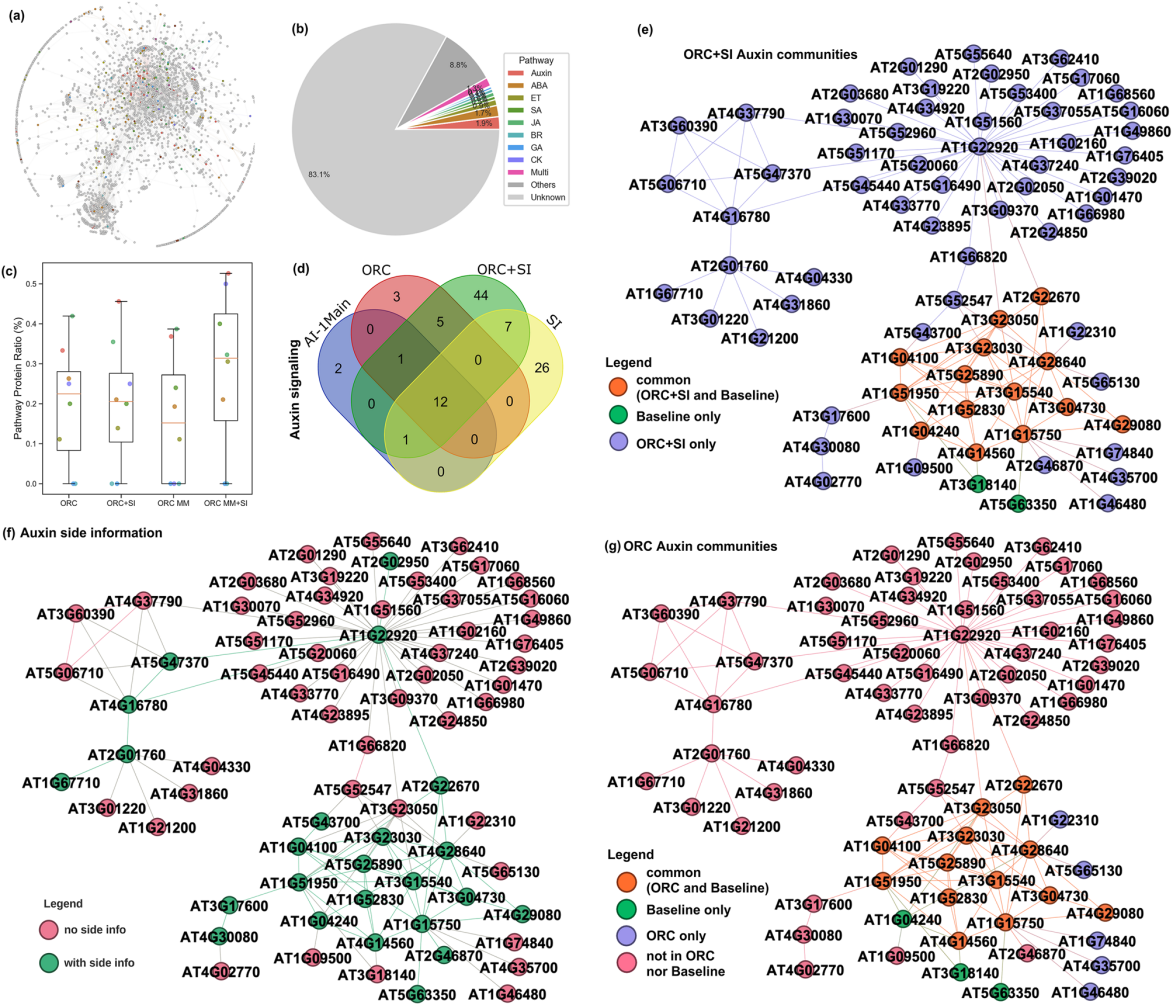
A comparison of network community detection methods for Arabidopsis phytohormone protein-protein interaction (PPI) ($\hbox {AI-1}_{{\mathrm{MAIN}}}$) network. **(a)** Network visualization with 2657 nodes and 5506 edges. Node colors represent known phytohormone pathway information (see color legend in **(b)**). **(b)** Ratio of phytohormone pathway proteins compared to the entire network. **(c)** A comparison of different community detection (CD) methods are shown for the percentage of identified pathway protein ratio. **(d)** Venn diagram of community overlaps between the baseline $\hbox {AI-1}_{{\mathrm{MAIN}}}$ auxin community with the largest auxin community identified by ORC final partition (*ORC* and *ORC+SI*) together with the side information list *SI*. **(e–g)** show the same network structure from the union of baseline $\hbox {AI-1}_{{\mathrm{MAIN}}}$ and *ORC+SI* largest auxin community. Node legend colors in **(e)** indicate the auxin side information, **(f)** indicate community memberships in the baseline and/or *ORC+SI*, and **(g)** indicate community memberships in the baseline and/or *ORC*.

### ORC-based community detection can identify phytohormone pathways in a whole-genome Arabidopsis protein network

We assessed the performance of the ORC-based methods on an even larger, more complex dataset in an attempt to further evaluate the approach. The Arabidopsis phytohormone signaling interactome dataset ($\hbox {PhI}_{{\mathrm{MAIN}}}$ and $\hbox {PhI}_{{\mathrm{FULL}}}$) analyzed above, was collected to over-represent phytohormone interactions. In order to address if we could identify major components of the phytohormone pathways from a genome-wide dataset collected under non-biased experimental conditions, we analyzed another larger protein–protein interactions data set. Here, we used the Arabidopsis Interactome version “main screen” ($\hbox {AI-1}_{{\mathrm{MAIN}}}$) network containing 5506 binary interactions between 2657 proteins^54^ (Fig. 5a). $\hbox {AI-1}_{{\mathrm{MAIN}}}$ shares 128 proteins (4.82%) with the literature-curated $\hbox {PhI}_{{\mathrm{MAIN}}}$ network and 405 proteins (15.24%) with the $\hbox {PhI}_{{\mathrm{FULL}}}$ network. From the $\hbox {AI-1}_{{\mathrm{MAIN}}}$ dataset, 188 proteins ($7.9\%$) are annotated as involved in the eight main phytohormone signaling pathways, 237 were annotated for a non-phytohormone function (8.9%), and a majority have an unknown function (83.2%, Fig. 5b). We used side information for 14.6% of the 2657 proteins in $\hbox {AI-1}_{{\mathrm{MAIN}}}$ (colored nodes in Fig. 5a), comprising both phytohormone and non-phytohormone annotations.

We first assessed the extent to which ORC-based community detection in the $\hbox {AI-1}_{{\mathrm{MAIN}}}$ can reveal the phytohormone signaling pathways. We observed around 40% of auxin and JA proteins and more than $20\%$ of ABA, CK, and SA proteins were successfully distinguished in their corresponding communities by four ORC-based methods in the genome-scale protein network (Fig. 5c). However, only 10% ET proteins but almost no GA or BR proteins were rediscovered by the four methods. This suggested the ORC-based community detection methods can still successfully identify some of the phytohormone pathways in a whole-genome scale Arabidopsis protein network but with varied sensitivity on detecting different phytohormone pathways. The average of the eight phytohormone pathway protein ratios is around $20\%$ for all four ORC-based methods, which is a significant reduction compared to their performance in $\hbox {PhI}_{{\mathrm{MAIN}}}$ and $\hbox {PhI}_{{\mathrm{FULL}}}$ networks (Figs. 3c, 4c,  5c). Such degradation in recovery of phytohormone proteins in the corresponding communities was expected since there is a significantly smaller proportion 7.9%) of annotated phytohormone proteins in $\hbox {AI-1}_{{\mathrm{MAIN}}}$ networks compared with 67.3% in $\hbox {PhI}_{{\mathrm{MAIN}}}$ and 30.2% in $\hbox {PhI}_{{\mathrm{FULL}}}$.

### Side information improves ORC-based community detection in a whole-genome Arabidopsis protein network

We next assessed whether the use of side information can assist the ORC-based community detection in the whole-genome scale protein network by evaluating the identified community size, number of motifs and modules, and robustness in detecting community contents. We found both *ORC* and *ORC+SI* identified similar sizes of communities and have comparable abilities in detecting the number of motifs (260 by *ORC* vs 253 by *ORC+SI*) and modules (141 by *ORC* and 139 by *ORC+SI*, Fig. S1a,b). Meanwhile, *ORC MM+SI* identified fewer motifs and more modules than *ORC MM* (Fig. S1b). However, *ORC* and *ORC+SI* identified more communities and relatively smaller community sizes compared to *ORC MM* and *ORC MM+SI*, suggesting that *ORC* final partition provides better resolution in community identification (Fig. S1b). Additionally, with a significant increase in network size, more large size communities were detected by all four methods, which was consistent with previous observations in $\hbox {PhI}_{{\mathrm{MAIN}}}$ and $\hbox {PhI}_{{\mathrm{FULL}}}$ networks (Fig. S1a). Given the size of $\hbox {AI-1}_{{\mathrm{MAIN}}}$ network, the small amount of side information as a proportion of the total network size ($14.6\%$) was not enough to significantly improve the community detection performance in terms of merging small motifs with shared functions into large modules.

To evaluate the detection robustness, we evaluated the conservation and variation of communities identified by four ORC-based methods with the originally defined 26 “baseline” communities identified by the edge-clustering method^54^. We defined the overlap (or conservation) ratio as the number of overlapping proteins in communities identified both by ORC-based methods and the edge-clustering method (Tables 1, 2, Fig. S2a). We defined variation as the difference in the community membership with respect to the edge-clustering method, where the variation was normalized by the community size. A normalized variation of 0 indicates all proteins identified by the ORC community are members of the baseline $\hbox {AI-1}_{{\mathrm{MAIN}}}$ community, while a normalized variation of 1 indicates *N* number of proteins belonging only to the ORC community, where *N* is the size of the $\hbox {AI-1}_{{\mathrm{MAIN}}}$ community (Table 2, Fig. S2b). We considered the communities with more than five times variation to the original $\hbox {AI-1}_{{\mathrm{MAIN}}}$ community as unlikely or untestable communities and did not include them in downstream analysis for interpretation. We found 7 and 11 such communities under *ORC MM* and *ORC MM+SI*, respectively but no such communities under either *ORC* or *ORC+SI* algorithms. In addition, the *ORC* and *ORC+SI* detected communities showed higher average overlap ratios ($0.77_{ORC}$ and $0.3_{ORC+SI}$, respectively, Fig. S2a, Table 2) and lower average variation ratios ($0.40_{ORC}$ and $0.51_{ORC+SI}$, respectively, Fig. S2b, Table 2) than *ORC MM* and *ORC MM+SI*, suggesting *ORC* algorithm is more robust than *ORC MM* in community detection on large-scale protein network. In particular, there are 7 highly conserved communities in the 26 baseline communities that were detected by all community detection methods with high conservation ratio and small variation ratio (Tables 1, 2, Fig. S2a,b). However, comparison between the two ORC-based algorithms revealed *ORC+SI* showed less robustness than *ORC* in terms of average conservation and variation ratios, suggesting the use of side information identified more communities with a relative higher variation level in community contents.

We hypothesized that the varied protein members uniquely belonging to an ORC community may contain novel components and relationships functioning in the pathway. An important application of community detection is to identify such novel components and relationships within known pathways, discover potential functional pathways, and reveal unknown interactions between pathways. To test our hypothesis and evaluate whether side information can assist ORC-based community detection to identify novel components and relationships in the whole-genome $\hbox {AI-1}_{{\mathrm{MAIN}}}$ dataset, we compared the shared and unique members between the phytohormone communities labeled in $\hbox {AI-1}_{{\mathrm{MAIN}}}$ network and detected communities by the ORC-based methods with and without side information. The $\hbox {AI-1}_{{\mathrm{MAIN}}}$ contains three “baseline” phytohormone communities, including Auxin signaling (16 proteins), Brassinosteroid signaling & phosphoprotein binding (10 proteins), and Seed germination and GA and JA signaling (7 proteins). Here, we focus on the Auxin signaling communities detected by ORC-based methods which showed the highest variation compared with the $\hbox {AI-1}_{{\mathrm{MAIN}}}$ baseline community (16 members, Fig. 5d–g, Tables 1,  2). By including the auxin pathway protein side information (Fig. 5e), we found more protein members in Auxin community detected by *ORC+SI* than the corresponding community detected by *ORC* (Fig. 5f,i). Based on the local community structure, we further divided the auxin community identified by *ORC+SI* into four sub-communities for illustration purposes: auxin-1 (bottom-right), auxin-2 (top-right), auxin-3 (top-left), and auxin-4 (bottom-left) (Fig. 5f). Auxin-1 is the core sub-community commonly identified by the baseline, *ORC* and *ORC+SI* methods (13 common proteins, Fig. 5d,f,g). While most of the auxin side information are found in auxin-1, we found 2 additional nodes with auxin side information present in each of the auxin-2,3,4 sub-communities (Fig. 5e). This resulted in the merging of the auxin-2,3,4 sub-communities with auxin-1 forming the extended auxin community discovered by *ORC+SI*. Specifically, the hub protein gene *AT1G22920* in auxin-2, which encodes sub-unit CSN5A of the COP9 signalosome (CSN)^55,56^ , provided crucial side information bridging auxin-2 with auxin-1 and auxin-3. This illustrates how side information can impact community detection.

To determine if this expanded auxin community is functionally relevant, GO enrichment analysis revealed that the community identified by *ORC+SI* was enriched for auxin function ($\hbox {Foldchange} = 19.66$, $P_{adj} = 2.06\mathrm{e}{-}05$) and CK function ($\hbox {Foldchange} = 28.55$, $P_{adj} = 1.14\mathrm{e}{-}02$), supporting the expanded community membership. When analyzing the biological functions for sub-communities by GO enrichment analysis, we found that auxin-1 was the main auxin-response community, impacting lateral root formation, and it was enriched in response to auxin GO term (Foldchange = 67.21, $P_{adj} = 4.63\mathrm{e}{-}25$). We did not find enriched GO biological functions in auxin-2 community. Previous research showed that the single csn5 mutants displayed reduced root growth in general, and a specific reduction in the response to auxin measured by root growth inhibition and lateral root emergence^57,58^. It is clear that CSN, and specifically the CSN5 sub-unit, plays diverse and pleiotropic roles in plant hormone signaling, defense, development and light signaling^57,59–61^. It has also been suggested that members of CSN form sub-complexes, which may serve specific functions, and evidence suggests that CSN5 does participate in these sub-complexes^57,62,63^. As such, the occurrence of AT1G22920/CSN5 as a major hub within the auxin-2 sub-community may reflect its multi-faceted role in the cell, and its connection between the other putative auxin sub-networks. Analysis of the individual proteins in auxin-2 suggested that it is involved in plant photomorphogenesis^57,58^. The auxin-3 sub-network is also connected to the CSN5A hub node, and GO enrichment analysis suggested shade avoidance function for auxin-3 (Foldchange >100, $P_{adj}$ = 2.41e−02). This phenomenon is controlled by multiple phytohormone pathways, including cytokinin signaling^64^. The crosstalk between cytokinin and auxin are well illustrated by the local network structure of auxin-4 that interacts with cytokinin via the hub protein encoded by AT2G01760 in this sub-community. This hub protein is annotated as a member of type-B response regulators (Response Regulator 14) involved in phosphorelay signal transduction acting upstream or within cytokinin-activated signaling pathway but without experimental validation^65^. Taken together, the use of side information helped identify potentially novel components in the auxin response pathway, linking to other possible functions and the cytokinin pathway. Future research to validate and explore these connections is needed.

**Table 1:** Sizes of overlapping communities between the $\hbox {AI-1}_{{\mathrm{MAIN}}}$ network and the ORC method results.

ID	AI-1MAIN baseline communities		All overlapping communities				The largest overlapping community			
			Community size (Number of communities)				Community size (Number of overlapped proteins)			
	Annotation	Size	ORC	ORC + SI	ORC MM	ORC MM + SI	ORC	ORC + SI	ORC MM	ORC MM + SI
4298	Water transport	11	11 (1)	11 (1)	11 (1)	11 (1)	11 (11)	11 (11)	11 (11)	11 (11)
1995	Transcription/gene expression	8	9 (1)	9 (1)	9 (1)	9 (1)	9 (8)	9 (8)	9 (8)	9 (8)
1534	Oxidoreductase activity	7	8 (1)	8 (1)	8 (1)	8 (1)	8 (7)	8 (7)	8 (7)	8 (7)
500	DNA binding	6	7 (1)	7 (1)	140 (1)	347 (1)	7 (6)	7 (6)	140 (6)	347 (6)
4167	Aromatic compound metabolism	6	7 (1)	7 (1)	7 (1)	7 (1)	7 (6)	7 (6)	7 (6)	7 (6)
5081	Ribonucloprotein complex	6	6 (1)	9 (1)	6 (1)	58 (1)	6 (6)	9 (6)	6 (6)	58 (6)
711	Cytoskeleton org. and root hair elong	9	15 (2)	11 (2)	35 (2)	19 (1)	9 (8)	9 (8)	9 (8)	19 (9)
5027	TCA cycle	8	11 (3)	10 (2)	18 (2)	27 (2)	5 (5)	7 (7)	15 (7)	24 (7)
1652	Auxin signaling	16	28 (4)	75 (3)	109 (2)	347 (1)	21 (13)	70 (14)	50 (15)	347 (16)
2535	Nucleosome assembly	13	24 (4)	29 (4)	30 (3)	29 (4)	8 (8)	10 (10)	11 (11)	10 (10)
5249	Potassium transport and kinase act	9	19 (4)	17 (4)	50 (1)	58 (1)	6 (6)	6 (6)	50 (9)	58 (9)
456	DNA repair and ubiquitination	8	12 (3)	12 (4)	140 (1)	60 (2)	7 (6)	6 (5)	140 (8)	59 (7)
899	Ubiquitination	7	8 (2)	8 (2)	159 (1)	269 (1)	5 (4)	5 (4)	159 (7)	269 (7)
1568	Transcription and nitrogen meta	7	36 (6)	24 (4)	275 (4)	361 (2)	3 (2)	5 (4)	140 (3)	347 (6)
4080	Calmodulin binding	9	13 (3)	13 (3)	13 (1)	13 (1)	5 (5)	5 (5)	13 (9)	13 (9)
4932	BR signaling and phosphoprotein bind	10	17 (4)	15 (4)	22 (2)	347 (1)	6 (5)	5 (5)	12 (9)	347 (10)
2796	RNA binding	10	15 (3)	13 (3)	121 (3)	21 (2)	8 (8)	5 (5)	8 (8)	9 (9)
3963	mRNA splicing	6	15 (4)	17 (4)	190 (2)	269 (1)	4 (3)	5 (3)	159 (5)	269 (6)
5255	Seed germ. and GB and JA signaling	7	29 (2)	43 (4)	59 (2)	323 (3)	8 (6)	6 (3)	8 (6)	9 (3)
4617	Ubiquitination	40	60 (10)	59 (12)	104 (7)	437 (7)	18 (17)	16 (15)	28 (27)	32 (28)
3347	No enriched GO annotations	14	36 (8)	51 (9)	159 (1)	269 (1)	10 (6)	9 (5)	159 (14)	269 (14)
4716	Transmembrane transport	350	526 (48)	533 (52)	790 (15)	1310 (20)	158 (158)	114 (114)	354 (315)	332 (306)
369	Ubiquitin-dependent degradation	6	17 (5)	17 (5)	299 (2)	328 (2)	3 (2)	3 (2)	140 (4)	59 (4)
1784	No enriched GO annotations	6	14 (3)	20 (4)	28 (2)	379 (3)	4 (4)	3 (2)	10 (5)	347 (3)
2706	No enriched GO annotations	22	58 (9)	108 (10)	148 (4)	484 (4)	9 (8)	8 (7)	46 (19)	77 (19)
1861	Vesicle trafficking	83	314 (27)	274 (26)	893 (13)	1186 (11)	16 (16)	16 (16)	107 (70)	89 (70)

**Table 2:** Overlap and variation ratio between $\hbox {AI-1}_{{\mathrm{MAIN}}}$ communities and ORC methods’ largest overlapping community.

ID	AI-1MAIN baseline communities		All overlapping communities				The largest overlapping community			
			Community size (Number of communities)				Community size (Number of overlapped proteins)			
	Annotation	Size	ORC	ORC + SI	ORC MM	ORC MM + SI	ORC	ORC + SI	ORC MM	ORC MM + SI
4298	Water transport	11	1.00 (1.00)	1.00 (1.00)	1.00 (1.00)	1.00 (1.00)	0.00 (0.00)	0.00 (0.00)	0.00 (0.00)	0.00 (0.00)
1995	Transcription/gene expression	8	1.00 (0.89)	1.00 (0.89)	1.00 (0.89)	1.00 (0.89)	0.12 (0.00)	0.12 (0.00)	0.12 (0.00)	0.12 (0.00)
1534	Oxidoreductase activity	7	1.00 (0.88)	1.00 (0.88)	1.00 (0.88)	1.00 (0.88)	0.14 (0.00)	0.14 (0.00)	0.14 (0.00)	0.14 (0.00)
500	DNA binding	6	1.00 (0.86)	1.00 (0.86)	1.00 (0.04)	1.00 (0.02)	0.17 (0.00)	0.17 (0.00)	22.33 (0.00)	56.83 (0.00)
4167	Aromatic compound metabolism	6	1.00 (0.86)	1.00 (0.86)	1.00 (0.86)	1.00 (0.86)	0.17 (0.00)	0.17 (0.00)	0.17 (0.00)	0.17 (0.00)
5081	Ribonucloprotein complex	6	1.00 (1.00)	1.00 (0.67)	1.00 (1.00)	1.00 (0.10)	0.00 (0.00)	0.50 (0.00)	0.00 (0.00)	8.67 (0.00)
711	Cytoskeleton org. and root hair elong	9	0.89 (0.89)	0.89 (0.89)	0.89 (0.89)	1.00 (0.47)	0.11 (0.11)	0.11 (0.11)	0.11 (0.11)	1.11 (0.00)
5027	TCA cycle	8	0.62 (1.00)	0.88 (1.00)	0.88 (0.47)	0.88 (0.29)	0.00 (0.60)	0.00 (0.14)	1.00 (0.07)	2.12 (0.04)
1652	Auxin signaling	16	0.81 (0.62)	0.88 (0.20)	0.94 (0.30)	1.00 (0.05)	0.50 (0.14)	3.50 (0.03)	2.19 (0.02)	20.69 (0.00)
2535	Nucleosome assembly	13	0.62 (1.00)	0.77 (1.00)	0.85 (1.00)	0.77 (1.00)	0.00 (0.62)	0.00 (0.30)	0.00 (0.18)	0.00 (0.30)
5249	Potassium transport and kinase act	9	0.67 (1.00)	0.67 (1.00)	1.00 (0.18)	1.00 (0.16)	0.00 (0.50)	0.00 (0.50)	4.56 (0.00)	5.44 (0.00)
456	DNA repair and ubiquitination	8	0.75 (0.86)	0.62 (0.83)	1.00 (0.06)	0.88 (0.12)	0.12 (0.29)	0.12 (0.50)	16.50 (0.00)	6.50 (0.02)
899	Ubiquitination	7	0.57 (0.80)	0.57 (0.80)	1.00 (0.04)	1.00 (0.03)	0.14 (0.60)	0.14 (0.60)	21.71 (0.00)	37.43 (0.00)
1568	Transcription and nitrogen meta	7	0.29 (0.67)	0.57 (0.80)	0.43 (0.02)	0.86 (0.02)	0.14 (1.67)	0.14 (0.60)	19.57 (0.03)	48.71 (0.00)
4080	Calmodulin binding	9	0.56 (1.00)	0.56 (1.00)	1.00 (0.69)	1.00 (0.69)	0.00 (0.80)	0.00 (0.80)	0.44 (0.00)	0.44 (0.00)
4932	BR signaling and phosphoprotein bind	10	0.50 (0.83)	0.50 (1.00)	0.90 (0.75)	1.00 (0.03)	0.10 (0.83)	0.00 (1.00)	0.30 (0.08)	33.70 (0.00)
2796	RNA binding	10	0.80 (1.00)	0.50 (1.00)	0.80 (1.00)	0.90 (1.00)	0.00 (0.25)	0.00 (1.00)	0.00 (0.25)	0.00 (0.11)
3963	mRNA splicing	6	0.50 (0.75)	0.50 (0.60)	0.83 (0.03)	1.00 (0.02)	0.17 (0.75)	0.33 (0.60)	25.67 (0.01)	43.83 (0.00)
5255	Seed germ. and GB and JA signaling	7	0.86 (0.75)	0.43 (0.50)	0.86 (0.75)	0.43 (0.33)	0.29 (0.12)	0.43 (0.67)	0.29 (0.12)	0.86 (0.44)
4617	Ubiquitination	40	0.42 (0.94)	0.38 (0.94)	0.68 (0.96)	0.70 (0.88)	0.03 (1.28)	0.03 (1.56)	0.03 (0.46)	0.10 (0.38)
3347	No enriched GO annotations	14	0.43 (0.60)	0.36 (0.56)	1.00 (0.09)	1.00 (0.05)	0.29 (0.80)	0.29 (1.00)	10.36 (0.00)	18.21 (0.00)
4716	Transmembrane transport	350	0.45 (1.00)	0.33 (1.00)	0.90 (0.89)	0.87 (0.92)	0.00 (1.22)	0.00 (2.07)	0.11 (0.10)	0.07 (0.13)
369	Ubiquitin-dependent degradation	6	0.33 (0.67)	0.33 (0.67)	0.67 (0.03)	0.67 (0.07)	0.17 (1.33)	0.17 (1.33)	22.67 (0.01)	9.17 (0.03)
1784	No enriched GO annotations	6	0.67 (1.00)	0.33 (0.67)	0.83 (0.50)	0.50 (0.01)	0.00 (0.50)	0.17 (1.33)	0.83 (0.10)	57.33 (0.01)
2706	No enriched GO annotations	22	0.36 (0.89)	0.32 (0.88)	0.86 (0.41)	0.86 (0.25)	0.05 (1.56)	0.05 (1.88)	1.23 (0.07)	2.64 (0.04)
1861	Vesicle trafficking	83	0.19 (1.00)	0.19 (1.00)	0.84 (0.65)	0.84 (0.79)	0.00 (4.19)	0.00 (4.19)	0.45 (0.12)	0.23 (0.15)

### Partial network observation impacts community detection

**Figure 6:**
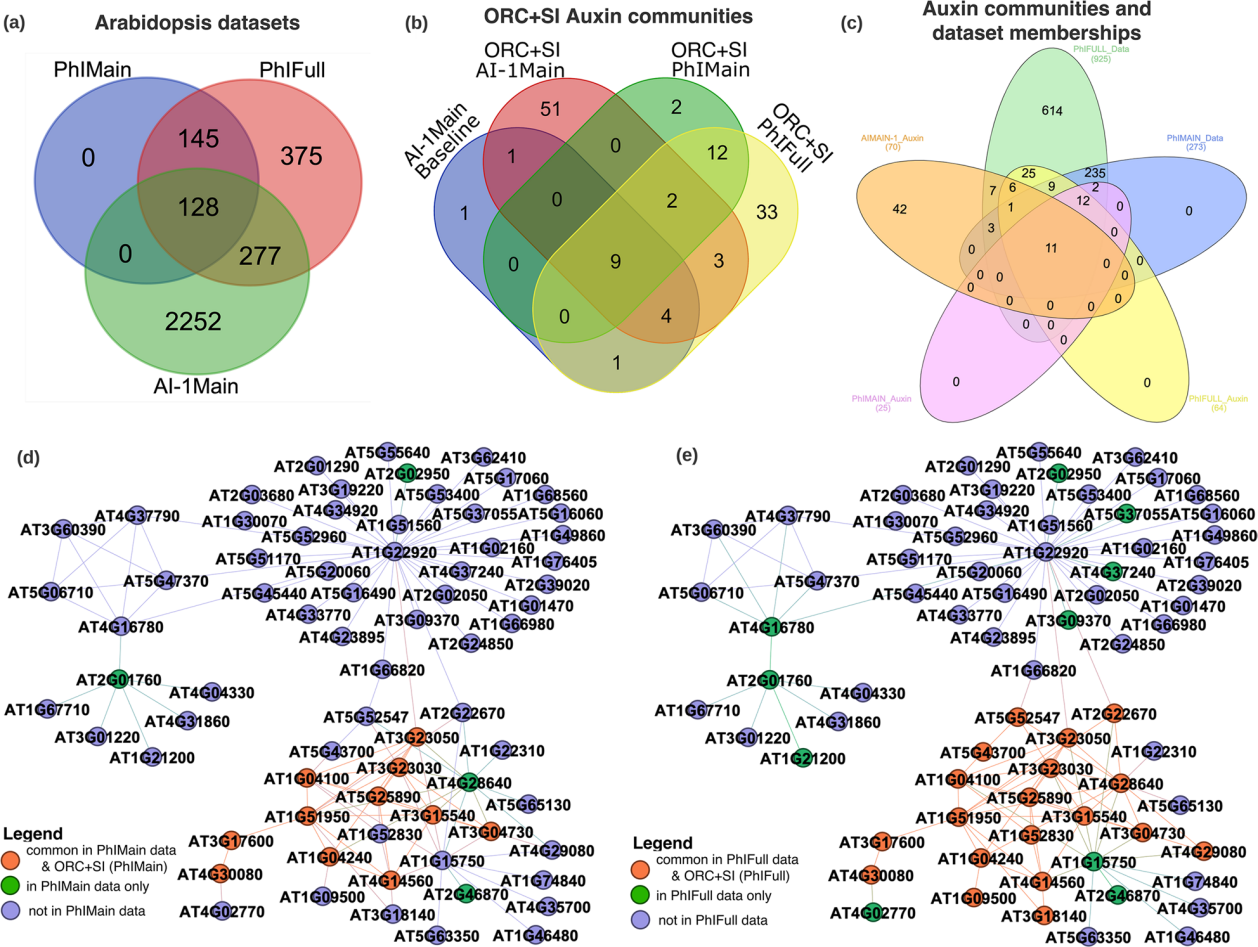
Auxin signaling pathway community overlap. **(a)** Venn diagram of overlap among the three Arabidopsis datasets. **(b)** Venn diagram showing the auxin *ORC+SI* community overlap across the three datasets: $\hbox {PhI}_{{\mathrm{MAIN}}}$, $\hbox {PhI}_{{\mathrm{FULL}}}$ and $\hbox {AI-1}_{{\mathrm{MAIN}}}$. **(c)** Venn diagram of auxin communities and dataset memberships. **(d,e)** Show the same network structure from the union of $\hbox {AI-1}_{{\mathrm{MAIN}}}$ baseline auxin community and the $\hbox {AI-1}_{{\mathrm{MAIN}}}$ *ORC+SI* largest auxin community. Legend colors in **(d)** indicate gene memberships and overlap in $\hbox {AI-1}_{{\mathrm{MAIN}}}$ baseline and $\hbox {PhI}_{{\mathrm{MAIN}}}$ *ORC+SI* largest auxin community, and **(e)** indicate gene memberships and overlap in $\hbox {AI-1}_{{\mathrm{MAIN}}}$ baseline and $\hbox {PhI}_{{\mathrm{FULL}}}$ *ORC+SI* largest auxin community.

Finally, we investigated how partial observation on networks impacted community detection in these real-world datasets. We used three Arabidopsis protein networks with varied network sizes to represent partial observation variation due to the sampling or assay sensitivity. $\hbox {AI-1}_{{\mathrm{MAIN}}}$ (2650 proteins) is considered to represent $\approx 2\%$ of the complete Arabidopsis $299,000 \pm 79,000$ (mean±SD) biophysical binary protein interactions^54^. The $\hbox {PhI}_{{\mathrm{MAIN}}}$ data (277 proteins) is a subset ($29.9\%$) of the $\hbox {PhI}_{{\mathrm{FULL}}}$ data (925 proteins), which is concentrated on phytohormone proteins and share 405 proteins with $\hbox {AI-1}_{{\mathrm{MAIN}}}$ (Fig. 6a). Between the $\hbox {PhI}_{{\mathrm{FULL}}}$ and $\hbox {AI-1}_{{\mathrm{MAIN}}}$ datasets, there are 375 proteins and 2252 proteins uniquely contained respectively. We compared the corresponding phytohormone communities for auxin, GA/JA, and BR, detected from the three Arabidopsis PPI-datasets, and compared them to the communities originally identified by edge-clustering method from the $\hbox {AI-1}_{{\mathrm{MAIN}}}$ dataset (Fig. 6b, Figs. S4a, S5a). Since we have shown from the previous sections that *ORC+SI* provided better community detection in $\hbox {AI-1}_{{\mathrm{MAIN}}}$, we used *ORC+SI* identified three phytohormone communities for comparison of the variation of protein members and local network structures (Fig. 6b–e, Figs. S3–S5). Comparison of protein member variation showed that partial observation resulted in 93, 54, and 48 non-detectable proteins in the auxin communities in $\hbox {PhI}_{{\mathrm{MAIN}}}$, $\hbox {PhI}_{{\mathrm{FULL}}}$, and $\hbox {AI-1}_{{\mathrm{MAIN}}}$, respectively (Fig. 6c). We found this was caused by a protein missing in a given dataset or broken connections (i.e., missing edges) between existing proteins and a detected auxin community (Fig. 6d,e, Fig. S3). For example, 93 non-detectable proteins in $\hbox {PhI}_{{\mathrm{MAIN}}}$ auxin community include 80 proteins missing in $\hbox {PhI}_{{\mathrm{MAIN}}}$ data and 13 proteins with broken links with detected auxin communities (Fig. 6c). In particular, four proteins present in $\hbox {AI-1}_{{\mathrm{MAIN}}}$ auxin community also were detected in $\hbox {PhI}_{{\mathrm{MAIN}}}$ data but failed to be detected in $\hbox {PhI}_{{\mathrm{MAIN}}}$ auxin community, including two proteins in auxin-1, one protein in auxin-2, one hub protein in auxin-4 (Fig. 6d). Likewise, there are 42 missing proteins and 12 broken-link proteins in the 54 non-detectable proteins in the $\hbox {PhI}_{{\mathrm{FULL}}}$ auxin community (Fig. 6c). Among 12 broken-link proteins, 10 of them spread across all four auxin sub communities in $\hbox {AI-1}_{{\mathrm{MAIN}}}$ (Fig. 6e). Visualizing the local network structure of the $\hbox {PhI}_{{\mathrm{FULL}}}$ auxin community shows that the missing proteins caused a change in edge connections among other existing proteins and thereby altered the network structure (Fig. S3b–d).

Similarly, partial observation caused dramatic community structure variation of the other two phytohormone communities, GA/JA and BR signaling communities, across the three datasets. Compared with $\hbox {AI-1}_{{\mathrm{MAIN}}}$, $\hbox {PhI}_{{\mathrm{FULL}}}$ identified more complete GA/JA and BR signaling communities, which is different from the results for the auxin community detection in $\hbox {AI-1}_{{\mathrm{MAIN}}}$ (Figs. S4, S5). For GA/JA community, *ORC+SI* identified a six-protein community in $\hbox {AI-1}_{{\mathrm{MAIN}}}$, a five-protein community in $\hbox {PhI}_{{\mathrm{MAIN}}}$, but a large community of 60 proteins in $\hbox {PhI}_{{\mathrm{FULL}}}$, containing 16 side information-labeled proteins in three subcommunities (Fig. S4a,b,d1). Compared with the $\hbox {PhI}_{{\mathrm{FULL}}}$ GA/JA community, relationship analysis and local network structure showed a majority of missing proteins and link-broken proteins in $\hbox {PhI}_{{\mathrm{MAIN}}}$ and $\hbox {AI-1}_{{\mathrm{MAIN}}}$ datasets (Fig. S4c,d). For BR community, *ORC+SI* identified a large community (51 proteins) in the $\hbox {PhI}_{{\mathrm{FULL}}}$ dataset, but failed to identify it in $\hbox {PhI}_{{\mathrm{MAIN}}}$, and only a small five-protein community in $\hbox {AI-1}_{{\mathrm{MAIN}}}$ due to majority of missing proteins and broken links in these two datasets (Fig. S5). Taken together, the relationship analysis and local community structure variation analysis for three phytohormone communities across three partial observed networks supports the hypothesis that protein (i.e., nodes) presence/absence variation between datasets causes significant differences in community detection. These results show that from a broad perspective, the level of network observation has a direct impact on community detection, where the loss of nodes through sampling techniques impairs the identification of functional communities and partial observation can obscure true community identification.

## Discussion

Biological systems are complex because they contain many individual components that are interconnected at different organizational scales. At the scale of cells, genes can be represented as biological networks (i.e., graphs) for the purpose of understanding the organization, structure and collective gene function for biological processes. However, complete biological network information is seldom available due to biological and technical limitations. Hence, partial network observation poses a problem in downstream community detection analysis where basic network science algorithms assume complete network information. Problems related to observability are not easily overcome, as they relate to technical limitations encountered when balancing high-throughput, large-scale, and accurate data collection of biological systems. To mitigate this limitation, we propose that using a subset of extensively studied proteins, whose biological function is documented, to guide community detection, we can still identify biologically functional communities from partially observed networks. Here, we introduced geometric topology-based network analysis for community detection and addressed the challenge of missing observations by incorporating nodal side information to improve the community detection performance.

Network geometry analysis has recently gained traction in network science, and offers new theoretical insights into the fundamental principles of complex systems based on their multiple scales of organization and information exchange^66^. In this work, we exploited the ORC-based community detection algorithm, which iteratively divides the network into communities by removing the edge with the most negative curvature based on the hypothesis that negative edge curvatures act as bridges between functional communities^27^. We further guided community detection by incorporating biological information in the form of functional gene annotation (i.e., nodal side information) to assist in edge removal criteria. We evaluated the community detection performance based on two ORC-based network community partitions: ORC final partition (*ORC*), and maximum modularity partition (*ORC MM*). From implementation, the *ORC* partition results in the finest-grained community partition while the *ORC MM* results in merged and larger communities. Synthetic network results without the aid of side information showed that *ORC* performed better at lower network observability ($\le 0.4$) than *ORC MM*, and vice versa for higher network observability ($\ge 0.4$, Fig. 2b,c). One possible reason for this is that for high network observability, *ORC* is penalized for dividing the ground truth community further into smaller constituent communities while for low network observability, the *ORC MM* is penalized for merging nodes from different ground truth memberships into one large community. As for the Arabidopsis PPI networks, the ORC final partition performed better compared to *ORC MM* in finding the building blocks such as smaller-sized communities and single-function modules, which are important in understanding the biological functions of the communities (Fig. 3e,f). For different application areas, finding the larger high-level subdivision of the network will be more relevant, and the *ORC MM* partition can be a more appropriate option.

In contrast to performing the basic community detection without side information, our results from both synthetic and real-world Arabidopsis PPI networks indicate that the ORC-based community detection coupled with side information improves community detection based on classification performance for the tested datasets (Figs. 2b,c, 3f, 4f) and baseline ($\hbox {AI-1}_{{\mathrm{MAIN}}}$) community comparison (Tables 1,  2) regardless of network size. The inclusion of side information affects *ORC* and the *ORC MM* implementation in a different manner. For *ORC*, the side information tends to merge small communities with shared functions into larger shared communities. This is observed especially in the synthetic networks and $\hbox {PhI}_{{\mathrm{MAIN}}}$ network where the *ORC+SI* identified significantly more single-function phytohormone communities. For the largest Arabidopsis ($\hbox {AI-1}_{{\mathrm{MAIN}}}$) PPI network, the *ORC+SI* provides the best balance between high overlap ratio and minimum variation compared with the baseline $\hbox {AI-1}_{{\mathrm{MAIN}}}$ communities. For *ORC MM*, the side information tends to break down the large communities into smaller communities as observed in $\hbox {PhI}_{{\mathrm{MAIN}}}$ and $\hbox {PhI}_{{\mathrm{FULL}}}$ networks. However, the choice of side information is very important. Wrong side information that does not correlate with ground truth community partition can negatively impact the community detection performance (Supplementary Fig. S6). This analysis shows that while the direct use of side information improves community detection performance, careful attention is required to ensure correct side information. To address this, the side information should be carefully curated based on high-confidence functional annotation from prior literature as ground-truth information. Alternatively, non-deterministic usage of side information is a possible extension to further improve the community detection performance without relying on a strong correlation assumption between side information and ground truth partition. This can be via weight adjustments of the probability mass distribution in the Wasserstein’s distance calculation. Additionally, side information together with a network generator inference framework^67^ can be used to aid reconstruction of the missing network^68^ due to partial observability.

Application of community detection on the three Arabidopsis protein networks reveals that the level of partial observation impacts the variation of presence and absence of a protein and its observed interactions with other proteins in a given network. This variation in turn impacts the detection of functional modules and motifs. As a case study, we focused on the comparison of the auxin communities across three datasets identified by ORC coupled with side information. Although the partially observed levels impact the protein community members and local auxin community structures across the three datasets, we observed its core protein network (denoted as auxin-1 sub-community, Figs. 5h,  6d,e) are conserved despite the variation of protein network sizes. Such stable local network content and structure suggest the partial observed protein networks due to the sampling approach do not impact the main auxin proteins and their relationships. Auxin is essential for plant cell growth by affecting cell division and cellular expansion, and it is also a general coordinator with multiple functions that interacts with several other phytohormone molecules to regulate plant growth and development by contributing to cell differentiation and specification^69,70^. The global interaction relationship of auxin proteins might be one of the reasons that results in the conserved structure. The varied protein members in communities from different datasets usually contain proteins involved in extensively interacting with other phytohormone pathways, post-translational modifications, and protein degradations^58,71–74^. For example, the interplay between COP9 signalosome and ubiquitin E3 ligase actively participates in phytohormone signaling pathways, including hormone perception and de-repression, through controlling over ubiquitin-proteasome-mediated protein degradation. The *ORC+SI* in this study identified two auxin sub-communities, auxin-1 with lateral root formation function and auxin-2 with photomorphogenesis function, that are associated with a hub protein CSN5A, a sub-unit of the COP9 signalosome (Fig. 5g)^55,56,58,75^. The biological functions of the two detected auxin communities by ORC-based algorithms are consistent with previous single-gene-mutant experiments. The protein members in auxin communities identified in this study can guide higher-order-mutants’ experiments for groups who are interested in auxin functions in plants. Furthermore, in planta validation of protein–protein interaction is required prior forward genetic experiments.

In summary, the ORC-based community detection coupled with side information can help identify biological meaningful modules from protein interaction networks regardless of size and complexity. While partial network observability significantly affects the content and structure of the network and hence the resulting detected communities, incorporation of the prior protein functional annotations as side information improves the ORC-based community detection performance, especially discovering potentially novel members and relationships associated to a community. Taken together, network geometric topology and side information can be exploited to understand the hierarchical organization of complex biological networks by detecting functional modules, discovering of novel molecules and interactions within a module, and revealing novel modules and their relationships. The new local network structure and communities reported here using the three Arabidopsis protein networks represent new hypotheses to be tested in future work. Additionally, areas of future exploration include the relaxation of the assumption on the strong correlation between side information and ground truth partition, further improvement of the community detection performance by considering non-deterministic methods of using side information, and reconstruction of partially observed networks based on side information and a network generator inference framework.

## Methods

### Synthetic network construction

We generated the synthetic networks using the Stochastic Block Model (SBM), which is a generative model for random graphs with planted community structures^41^. The planted community structures are considered as ground truth community assignments used for evaluating the performance of community detection methods. The generated synthetic networks contain a pre-defined number of communities of varying chosen sizes and intra- and inter-community edge linking probabilities. The complete network is denoted by *G*(*V*, *E*) with *V* denoting the set of nodes and *E* denoting the set of edges. The network size is $n=|V|$ which is the total number of nodes. The intra- and inter-community edge linking probabilities are denoted by $p_{in}$ and $p_{out}$, respectively. The observed network, denoted as $G_{obs}(V_{obs},E_{obs})$, is a subgraph of G where only a subset of nodes $n_{obs}$ are observable. For the synthetic network experiment, we randomly sample a percentage of the total nodes as observable starting at 10% observability. We re-run the test for increasing values of observability percentages up to the full 100% observability, i.e., $n_{obs}=[0.1, 0.2, \ldots , 1]\times n$. Similarly, we also generate a subset of nodes $n_s$ randomly selected from the observed nodes in $n_{obs}$ in which the community ground truth labels are pre-defined. We define this set of observed nodes with known community labels (or a priori side information) as *S*, with $n_s=|S|$ denoting the number of nodes with known side information. In these experiments, we also vary the size of *S* and evaluate the ORCCI performance. We test this for $n_s=[0, 0.1, \ldots , 1]\times n_{obs}$. The performance is quantified by the normalized mutual information (NMI) which is a common metric use to evaluate community detection performance.

For the synthetic network experiment, we ran the community detection on an SBM model of 1000 nodes with 10 pre-defined ground truth communities of varying sizes [150, 125, 125, 110, 100, 100, 90, 75, 75, 50]. Figure 2a shows the network visualization with color-coded ground-truth labels. We perform the community detection and evaluate the performance for all combinations of network observability sizes $n_{obs}$ and side information sizes $n_s$. Finally, we repeat the experiments on all 20 different instances of the SBM generated network using the same network configuration.

### Ollivier-Ricci curvature

The proposed community detection algorithm utilizes the coarse geometry topological concept of the Ollivier-Ricci curvature^20,76^ to graphs or networks^77^. The notion of curvature is a measure of the amount by which a geometric object deviates from being flat (Euclidean plane). The idea of Ricci curvature, on the other hand, is a notion of curvature to a discrete triangulated surface (coarse geometry) or graphs. In the graph/network context, the ball around *x* is the set of neighbors of *x* (same for *y*). Similarly, the idea is to find the optimal way to transfer the ball of mass from the vertex *x* to *y*.

### ORCCI community detection and side information

Algorithm 1 shows the implementation of the proposed community detection augmented with side information. The main idea is the recursive removal of the most negatively curved edge in the network while keeping in mind not to remove the “known” edges based on the a priori side information. Note that this a priori side information is assumed to be carefully curated from high-confidence functional annotation from prior literature. The algorithm performance will be limited if the side information is not correlated with the hidden community structure.
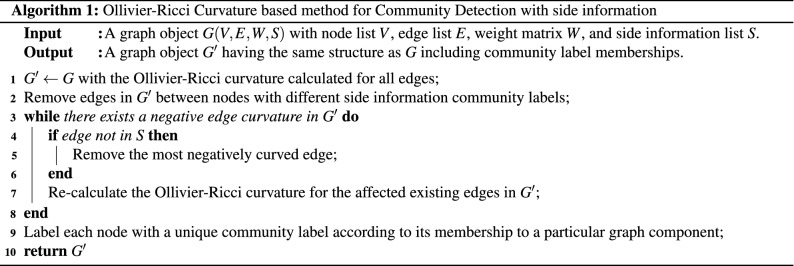


### Complexity

The time complexity of the proposed ORC-based community detection algorithm boils down to the calculation of the edge ORC of the network. The time complexity to compute the ORC for each edge is essentially the Wasserstein distance computation complexity based on linear programming. Practical run time complexity using network (transportation) simplex algorithm^78^ was shown to be super-cubic. Interior-point or Orlin’s algorithms have complexity of $O(V^3\log V)$, with *V* as the total number of vertices in the Wasserstein distance sub-problem^79,80^ (note that *V* depends on twice the average degree of the network typically with $V\ll N$ and $V\ll E$). In the worst case, cycling through each network edge and re-calculating all existing affected edges lead to $O((EV)\cdot V^3\log V)$. Strategies can be utilized to improve the computation complexity of the proposed algorithm either via a wavelet EMD approximation^79^ of the Wasserstein distance or an ORC bounds analysis^81^. The Wasserstein distance computation can be improved from $O(V^3\log V)$ to *O*(*V*) via the wavelet EMD approximation leading to an overall time complexity of $O(EV^2)$ for the proposed algorithm.

### Arabidopsis protein interactome networks

We apply the ORCCI-based community detection methods to the real-world biological protein networks to discover functional motifs and modules. This can allow us to better understand the protein dynamics and evolution and potentially discover novel insights. We choose three publicly available protein interaction networks that map the pairwise physical associations between proteins in the chosen model plant *Arabidopsis thaliana* (Arabidopsis)^47^. Arabidopsis, as a model organism in plant biology, has a large number of genes within its genome that are functionally identified and characterized by classical molecular and genetic approaches. For example, several databases such as TAIR are available with gene functional annotation and several of mutation lines uncovering the genotype-phenotype relationships. This makes it a gold-standard platform to explore the biological networks^82^.

We choose the three protein interaction data sets for the following reasons: (a) the datasets share a core set of proteins that are involved in phytohormone signaling associated pathways, and (b) the datasets vary in network sizes. For example, the Arabidopsis phytohormone interactome main ($\hbox {PhI}_{{\mathrm{MAIN}}}$) network with 273 nodes and phytohormone interactome full ($\hbox {PhI}_{{\mathrm{FULL}}}$) network with 926 nodes serve as the small and medium data sets in this study^47^ (see Supplemental Tables 1, 2). Both Arabidopsis protein networks are derived from the same study that focuses on the phytohormone signalling pathways which integrate external stimuli or internal cues to regulate plant biological processes such as growth, development, and response to stress. Arabidopsis Interactome version 1 “main screen” ($\hbox {AI-1}_{{\mathrm{MAIN}}}$^54^, 2661 nodes) is the genome-wide protein network that allows us to observe the local networks and generate systems-level hypothesis (Supplemental Table 3). Topological analysis reveals that the three networks exhibit a hierarchical network structure, which integrates a scale-free topology with an inherent modular structure that the sparsely connected sub-networks are connected to highly clustered neighbourhoods by a few of hub nodes^5^ (Figs. 3a, 4a, 5a). The hierarchical organization of the three protein networks indicates both the relationship between two proteins and the communications among sub-networks, including the functional motifs and modules.

Based on the primary topological structure analysis, we perform the ORC-based community detection coupled with a priori node side information on the three Arabidopsis protein networks to identify functional motifs and modules. We compare our proposed method, the ORC-based community detection evaluated at the final partition (ORC) and maximum modularity partition (ORC MM), and the baseline method, the betweenness centrality method (Btwns) used in Ref.^47^ for the $\hbox {PhI}_{{\mathrm{MAIN}}}$ network analysis. We quantify the accuracy of different community detection methods by comparing the biological functions of the predicted communities to the ground truth of the biological function and sub-cellular locations of proteins retained in the corresponding community. The conserved core set of nodes and varied sizes among the three Arabidopsis protein networks allow us to apply the proposed community detection method and evaluate how varied levels of observability and side information influence the prediction of functional motifs and modules in biological networks with increased size and complexity.

### Protein–protein interaction datasets and gold standards

In contrast to the ground-truth data used as gold standards for verifying the community detection in the synthetic networks, there is rarely a straightforward ground-truth for biological networks or modules. To evaluate the performance of different community detection methods on the protein–protein network, we collected the meta-data for individual proteins that involve in eight phytohormone pathways. We considered several compensate gold standards derived from the gene set analysis, including the Kyoto Encyclopedia of Genes and Genomes (KEGG) pathway analysis^45^ and Database for Annotation, Visualization and Integrated Discovery (DAVID)^52^, gene ontology enrichment analysis^46,53^, and gene description analysis.

## Supplementary Information

Supplementary Information.

Supplementary Table 1.

Supplementary Table 2.

Supplementary Table 3.

Supplementary Table 4.

Supplementary Table 5.

Supplementary Table 6.

## Data Availability

Codes available at: https://github.com/jcsia/orcci.
