# Supplementary material for: Inferring functional communities from partially observed biological networks exploiting geometric topology and side information
Source: Sci Rep. 2022 Jun 27;12:10883. doi: 10.1038/s41598-022-14631-x (PMC9237089; doi:10.1038/s41598-022-14631-x)
Supplement: Supplementary file 1 — Supplementary Information. [file 41598_2022_14631_MOESM1_ESM.pdf]

Supplementary Information

Related work discussion and clarifications about community detection A key challenge in cell biology is to uncover the organizing principles that control the interactions of all molecular components in a cell, which shape the structure and function of a living cell. Cellular biological networks describe such physical or functional interactions among DNA, RNA, proteins, and small molecules. These interactions can be directly measured through experiments or indirectly measured by computational methods. For example, the physical interactions among proteins can be experimentally captured by yeast-two-hybrid assay[83] and tandem affinity purification or computationally by domain or ortholog interactions, gene fusion and phylogenetic profiles. Functional interactions among genes and their regulatory elements can be inferred by many statistical methods, such as the epistatic interaction between two genes in a genetic network and the regulatory relationships in a gene regulatory network. The identification of hierarchical organization and its corresponding functional motifs and modules in a biological network faces two main challenges: (i) The high-dimensionality and complex multi-scale interdependence among biological constituents renders current techniques as inaccurate or providing incomplete understanding. (ii) The overlapping functions and crosstalk pathways as well as the lack of ground truth information calls for new mathematical approaches to decipher the complexity of biological networks.

Identifying community structures in a network, also called community detection or graph clustering, is a classic problem in network science. In this area of study, a network is a model abstraction of a system composed of interacting entities denoted as nodes, and binary interactions denoted as edges. For the biological network case, these entities are the interacting genes, proteins or molecules. The identification of community structures reveals insight on how the network is organized, potentially revealing distinct properties unique to this subset of nodes. While there is no strict definition of what a network community is, accepted convention defines a network community as a set of nodes that are internally densely connected while externally sparsely connected to other nodes not belonging to the same set[6]. In biological network, these tightly connected set of nodes often corresponds to the biologically relevant functional modules. There have been many network community detection methods proposed in the last decade that utilize different techniques and network metrics to find the best community partition of the network[6], e.g., the Girvan-Newman (GN) method is based on the edge betweenness which is a measure of the number of shortest paths passing through an edge, and the modularity-based community detection methods maximizes the modularity metric which is a measure of the quality of the division of the network into densely connected clusters. The limitation of the GN method is on its computational complexity, while the limitation of modularity-based methods is on the resolution limit of the community detection where it fail to identify communities smaller than a scale which depends on the size of the network[84]. In this paper, we will utilize a network geometry-based community detection method that is based on the network curvature. The Ollivier-Ricci curvature (ORC)-based community identification (ORCCI) is a method that iteratively removes the most negative curvature edge until the network is divided into distinct communities comprising only of non-negative curvature edges. This is because the sign of the edge curvatures gives insight on the local community structure: positive ORC edges are well connected and naturally form a community, while negative ORC edges could be interpreted as “bridges” between communities[27].

Most of the community detection algorithms assumes a complete network information in order to reliably provide an accurate community partition. However, as in many real-world problems, we do not always have a complete picture of the entire network structure, i.e., not all nodes and/or edges are available. Especially for biological networks, acquiring a complete picture of the entire network is typically impossible. This is not only due to limitations in data collection but also in resource constraints, data size and experiment execution time. Therefore, only a partial snapshot of the complete network is typically available for data analysis. Performing community detection on such partially observed networks with missing network information can lead to inaccurate community identification. Despite the loss of some network information, we normally have an insight to some of the nodes and their relationships in a community depending on the network application. For the biological context, based on previous studies, we may know that certain genes/proteins involving in a biological pathway responsible for a particular biological function. We call these insights as a priori node (or edge) side information. Using these side information in the community detection procedure can improve the overall community detection performance in spite of only having a partial observation of the network.

Here, we utilize the Ollivier-Ricci curvature-based community detection algorithm by integrating the network topological structure and a priori biological functional data. We first validate the algorithm on synthetic network data by varying levels of network observability and side information. We then apply the method to three real-world Arabidopsis protein networks to identify the essential pathways and communities in partially observed biological networks with scarce a priori gene functional information. The three Arabidopsis protein networks vary in network sizes but share a core set of proteins involved in phytohormone signaling pathways. We quantify the accuracy by comparing the biological functions of the predicted communities to the ground truth of individual proteins. This analysis identified the conserved and varied modules across three Arabidopsis protein networks. Our work suggests ORC-based community detection coupled with side information can be used to explore the novel modules and relationships in complicated biological networks, which sheds light on biological network dynamics and evolution.

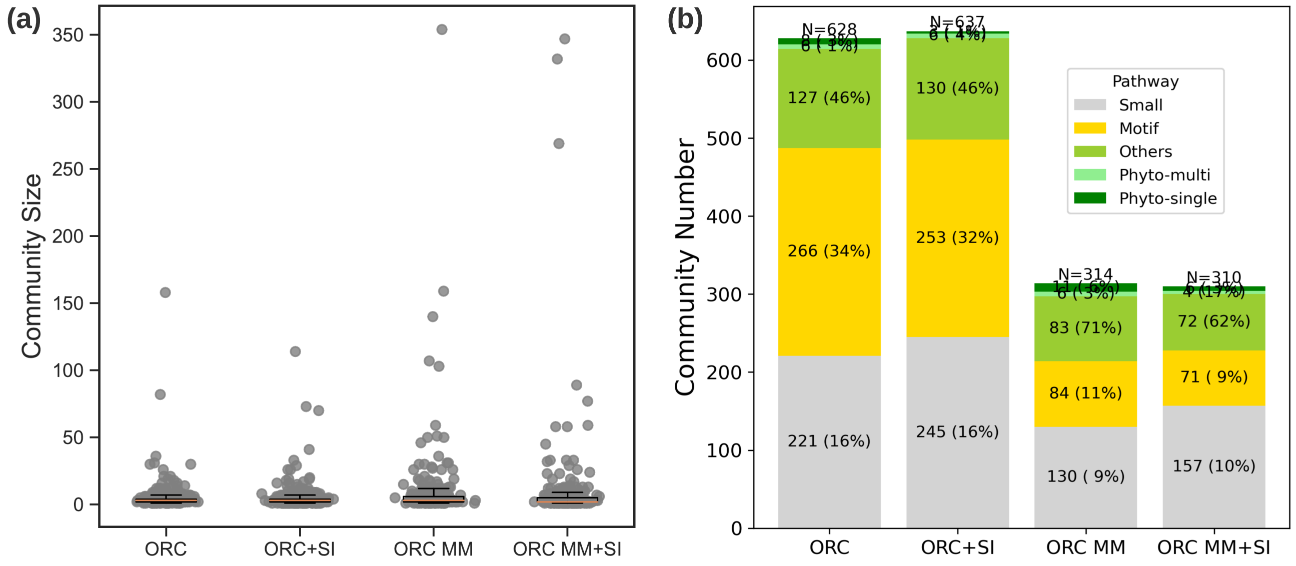

Figure S1. A comparison of network community detection methods for Arabidopsis phytohormone protein-protein interaction (PPI) (AI-1_{MAIN}) network based on **(a)** community size distribution and (**b**) community number and functional decomposition.

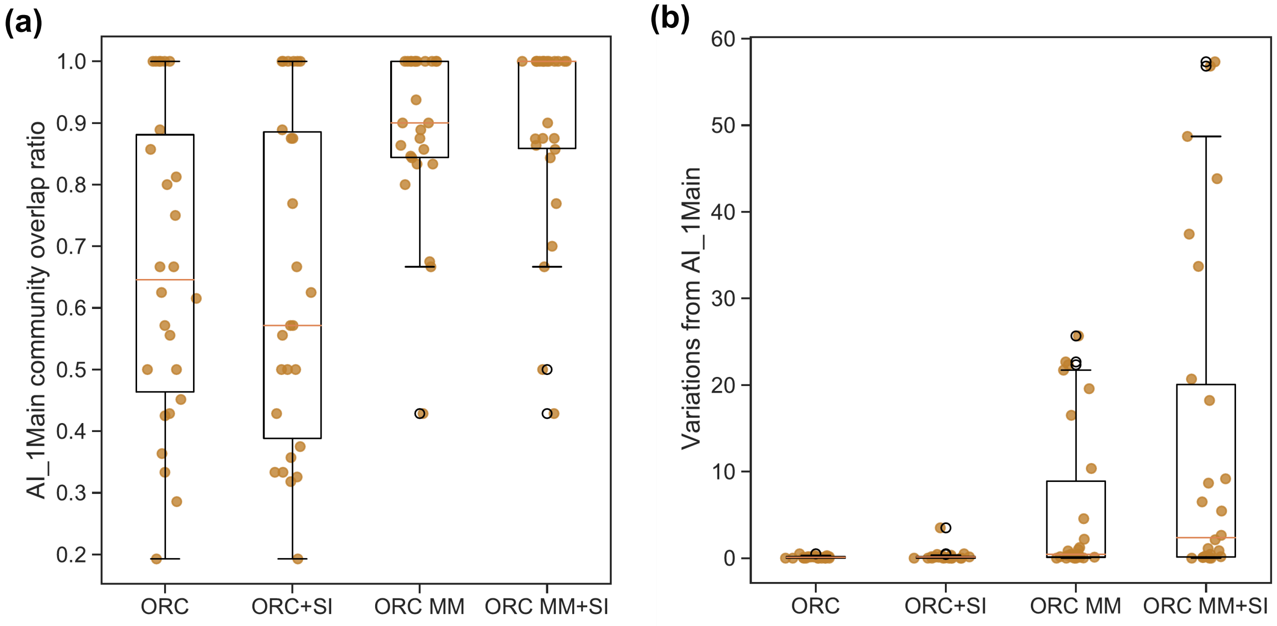

Figure S2. A comparison of network community detection methods for Arabidopsis phytohormone protein-protein interaction (PPI) (AI-1_{MAIN}) network based on **(a)** overlap (or conservation) ratio and **(e)** normalized variations with respect to AI-1MAIN communities.

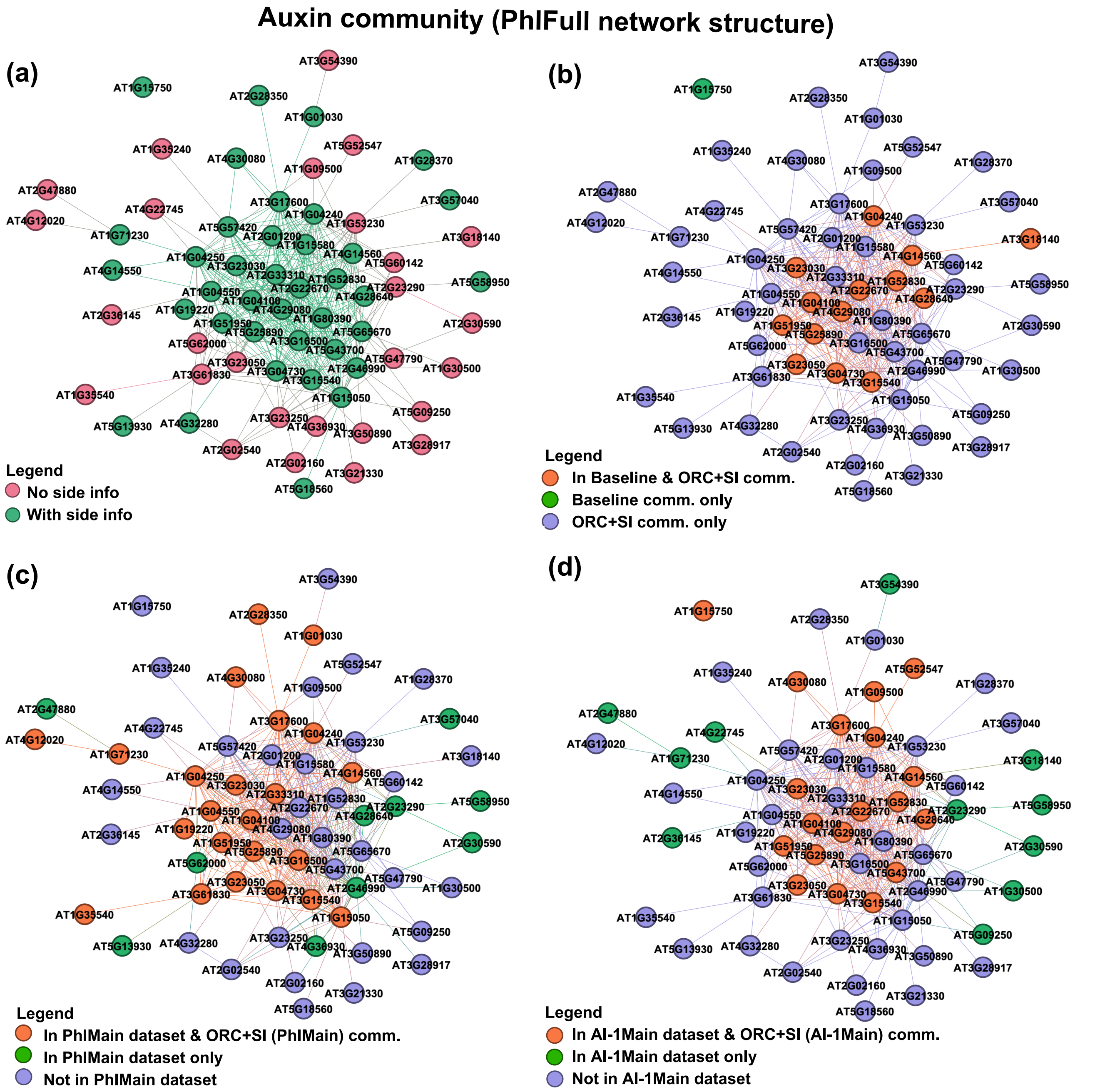

Figure S3. **Auxin signaling pathway community and dataset memberships.** **(a)**-**(d)** show the same network structure from the union of AI-1_{MAIN} baseline auxin community and the PhI_{FULL} *ORC+SI* largest auxin community. Legend colors in **(a)** indicate node side information, **(b)** indicate node membership in AI-1_{MAIN} baseline and PhI_{FULL} ORC+SI largest auxin communities, **(c)** indicate membership in PhI_{FULL} largest auxin community and PhI_{MAIN} dataset, and **(d)** indicate membership in PhI_{FULL} largest auxin community and AI-1_{MAIN} dataset.

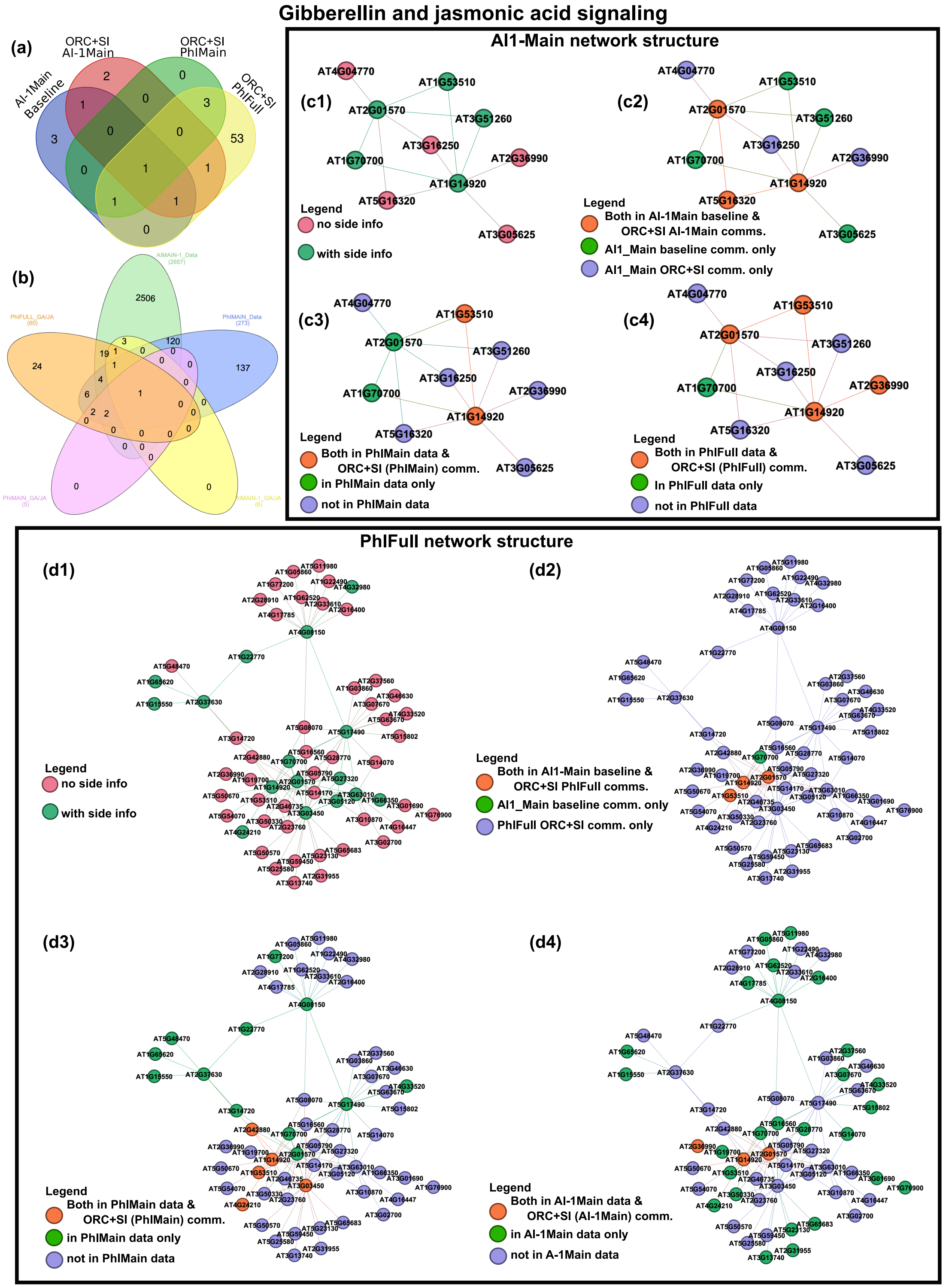

Figure S4. Seed germination and gibberellin and jasmonic acid signaling pathway (GA/JA) community and dataset memberships. **(a)** Venn diagram showing the GA & JA *ORC+SI* community overlap across the three datasets: PhI_{MAIN}, PhI_{FULL} and $\text{AI-1}_{\text{MAIN}}$. **(b)** Venn diagram of GA & JA communities and dataset memberships. The same network structures are shown in **(c)** from the union of $\text{AI-1}_{\text{MAIN}}$ baseline GA & JA community and the $\text{AI-1}_{\text{MAIN}}$ *ORC+SI* largest GA & JA community and **(d)** from the union of $\text{AI-1}_{\text{MAIN}}$ baseline GA & JA community and the PhI_{FULL} *ORC+SI* largest GA & JA community. Legend colors in **(c)** and **(d)** indicate **(1)** side information, **(2)** community overlap, **(3)** membership in PhI_{MAIN} dataset, and **(4)** membership in $\text{PhI}_{\text{FULL}}/\text{AI-1}_{\text{MAIN}}$ dataset.

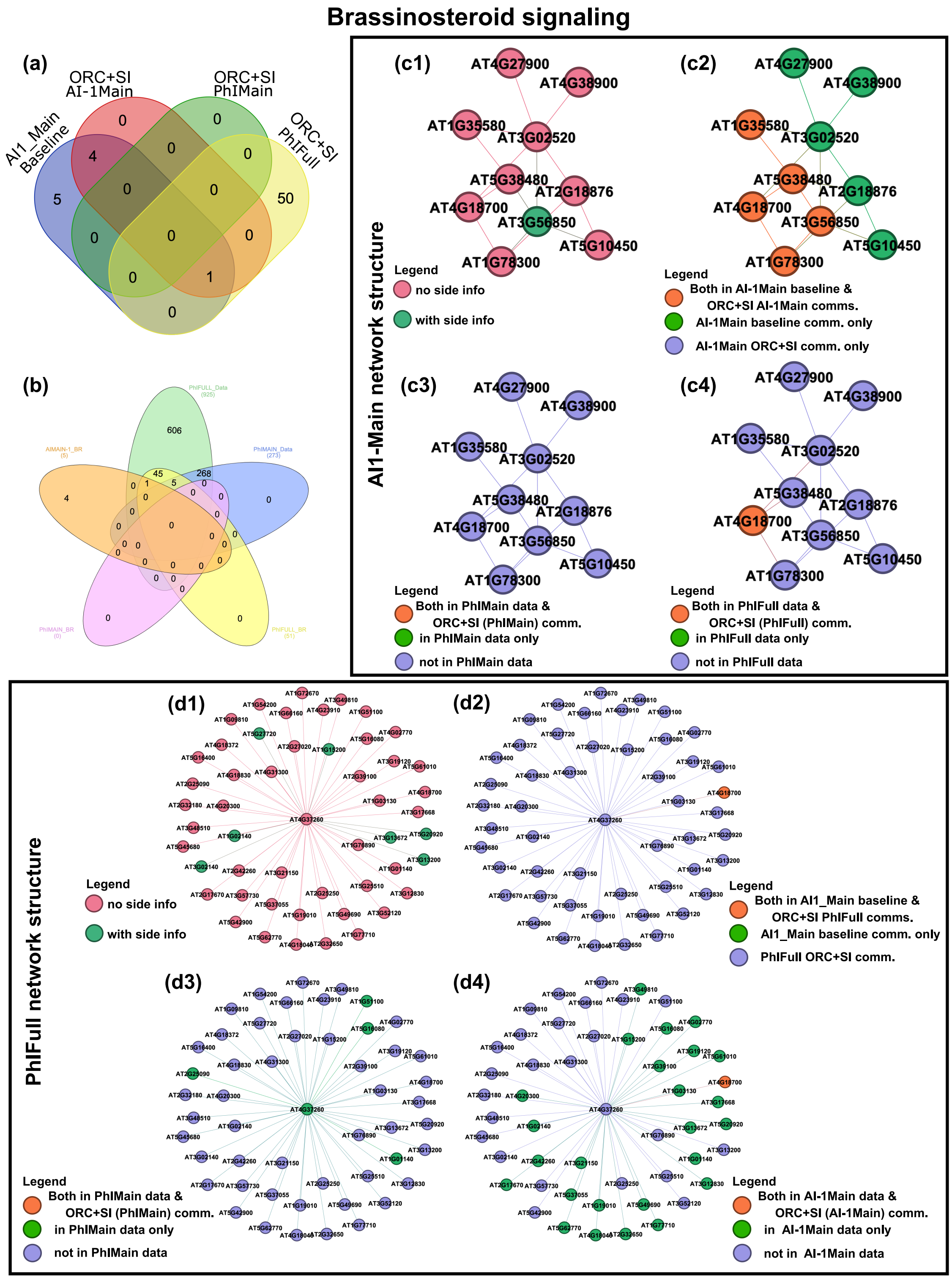

Figure S5. Brassinosteroid signaling pathway (BR) community and dataset memberships. **(a)** Venn diagram showing the BR *ORC+SI* community overlap across the three datasets: $\mathrm{PhI_{MAIN}}$, $\mathrm{PhI_{FULL}}$ and $\mathrm{AI\text{-}1_{MAIN}}$. **(b)** Venn diagram of BR communities and dataset memberships. The same network structures are shown in **(c)** from the union of $\mathrm{AI\text{-}1_{MAIN}}$ baseline BR community and the $\mathrm{AI\text{-}1_{MAIN}}$ *ORC+SI* largest BR community and **(d)** from the union of $\mathrm{AI\text{-}1_{MAIN}}$ baseline BR community and the $\mathrm{PhI_{FULL}}$ *ORC+SI* largest BR community. Legend colors in **(c)** and **(d)** indicate **(1)** side information, **(2)** community overlap, **(3)** membership in $\mathrm{PhI_{MAIN}}$ dataset, and **(4)** membership in $\mathrm{PhI_{FULL}}$/$\mathrm{AI\text{-}1_{MAIN}}$ dataset.

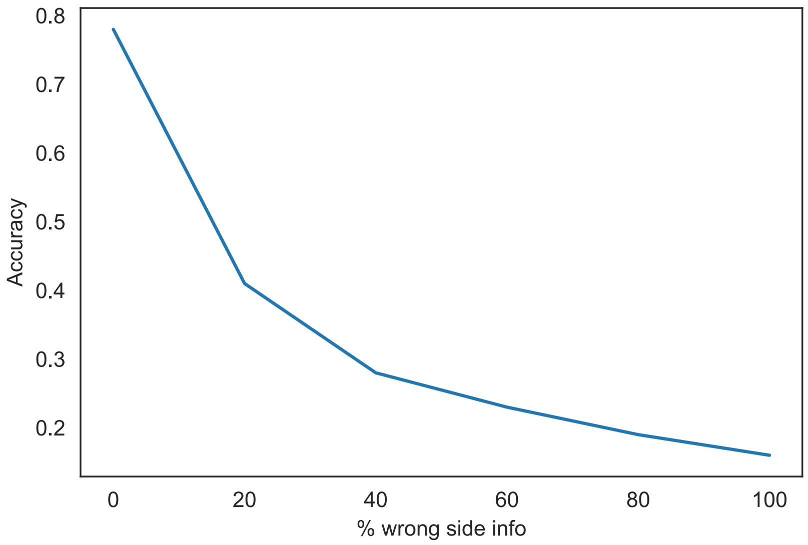

Figure S6. **PhI$_{\text{MAIN}}$ network wrong side information analysis on *ORC+SI*.**

Supplemental Table 1. **Protein and community annotation in *Arabidopsis thaliana* phytohormone interactome main (PhI$_{\text{MAIN}}$) network.**
Supplemental Table 2. **Protein and community annotation in *Arabidopsis thaliana* phytohormone interactome full (PhI$_{\text{FULL}}$) network.**
Supplemental Table 3. **Protein and community annotation in *Arabidopsis thaliana* interactome version 1 "main screen" (AI-1$_{\text{MAIN}}$) network.**
Supplemental Table 4. Summary of functional motifs and modules detected from three *Arabidopsis thaliana* protein interactome networks.
Supplemental Table 5. Summary of the network traits for the three *Arabidopsis thaliana* protein interactome networks.
Supplemental Table 6. Summary of the network traits of the largest auxin phytohormone community identified by *ORC+SI* among the three *Arabidopsis thaliana* protein interactome networks.

Consequences of having incorrect side information on network community identification In the main manuscript, we show that side information improves community detection in both synthetic and real-world biological network case studies. However, this assumes that the side information list used in assisting the community detection methods are correlated to the hidden network community structure. Here, we investigate the impact of supplying incorrect side information to community prediction performance. We use the PhI$_{\text{MAIN}}$ network and modify the labels for a subset of nodes in the original side information list. We then evaluate the community detection performance by varying the number of incorrect side information.

Supplemental Fig. S6 shows the community detection accuracy versus varying percentage of incorrect SI. The n number of incorrect SI nodes are selected from the top n highest degree nodes from the original list of nodes with side information. Results show that the number of incorrect SI nodes negatively impacts the accuracy with around 35% decrease in accuracy resulting from 20% incorrectly chosen SI. This analysis shows that while SI improves community detection performance, careful attention must be given to the correctness of these SI labels especially to "important" nodes. To avoid assigning incorrect SI, the SI should be carefully curated based on high-confidence functional annotation from prior literature. As argued in[51], metadata are not the same as ground truth. Hence, careful attention must be given in creating the side information list to avoid errors that could impact the overall community detection performance.

Comparison of network traits of communities sharing GO terms Supplemental Table 5 summarizes the network traits for the three *Arabidopsis* protein interactome networks. Here, we specifically discuss some of the community network traits for the largest auxin phytohormone community as identified by *ORC SI* (Supplemental Table 6). The average degree for the largest auxin community are 5.08, 15.20, and 6.36 from PhI$_{\text{MAIN}}$, PhI$_{\text{FULL}}$ and AI-1$_{\text{MAIN}}$ networks, respectively. This shows that the three networks are different in terms of structure. While PhI$_{\text{MAIN}}$ and PhI$_{\text{FULL}}$ are both phytohormone-focused networks, PhI$_{\text{FULL}}$ has a larger and expanded network. This explains PhI$_{\text{FULL}}$ having a larger average degree; therefore,

more observed interaction between phytohormone nodes as captured by the network. Meanwhile, AI-1_{MAIN} network is not a dedicated phytohormone network. While AI-1_{MAIN} has the largest structure among the three networks, the lower average degree compared to PhI_{FULL} for the auxin community shows that there are less observed auxin interactions in the network. This is similarly captured in the average clustering coefficients with values 0.18, 0.40, 0.10 for the PhI_{MAIN}, PhI_{FULL} and AI-1_{MAIN} networks, respectively. Meanwhile, the average betweenness centrality are 242, 1398, 9087 for the PhIMain, PhIFull and AI-1_{MAIN} networks, respectively. Betweenness centrality measures the extent to which a vertex lies on paths between other vertices. The dramatic increase in average betweenness centrality as the network size increases also indicates some sense of network sparsity. All other network measures for other phytohormone communities can be extracted from Supplemental Table 5.
